# miR-30e-5p-mediated FOXD1 promotes cell proliferation by blocking cellular senescence and apoptosis through p21/CDK2/Rb signaling in head and neck carcinoma

**DOI:** 10.1038/s41420-023-01571-2

**Published:** 2023-08-10

**Authors:** Tong Wu, Zhongyuan Yang, Weichao Chen, Mingjie Jiang, Zhichao Xiao, Xuan Su, Zan Jiao, Yongchao Yu, Shuwei Chen, Ming Song, Ankui Yang

**Affiliations:** 1 Department of Head and Neck Surgery, Sun Yat-sen University Cancer Center, Guangzhou, 510060 China; 2 State Key Laboratory of Oncology in Southern China, Guangzhou, 510060 China; 3 Collaborative Innovation Center of Cancer Medicine, Guangzhou, 510060 China; 4 Department of Otolaryngology-Head Neck Surgery, Loudi Central Hospital, Loudi, Hunan Province China

**Keywords:** Oral cancer, Cell growth

## Abstract

Forkhead box D1 (FOXD1) belongs to the FOX protein family, which has been found to function as a oncogene in multiple cancer types, but its role in head and neck squamous cell carcinoma (HNSCC) requires further investigation. Our research aimed to investigate the function of FOXD1 in HNSCC. Bioinformatics analysis indicated that mRNA level of FOXD1 was highly expressed in HNSCC tissues, and over-expressed FOXD1 was related to poor prognosis. Moreover, FOXD1 knockdown increased the ratio of senescent cells but decreased the proliferation ability, while FOXD1 overexpression obtained the opposite results. In vitro experiments revealed that FOXD1 bound to the p21 promoter and inhibited its transcription, which blocked the cyclin dependent kinase 2 (CDK2)/retinoblastoma (Rb) signaling pathway, thus preventing senescence and accelerating proliferation of tumor cells. CDK2 inhibitor could reverse the process to some extent. Further research has shown that miR-3oe-5p serves as a tumor suppressant by repressing the translation of FOXD1 through combining with the 3’-untranslated region (UTR). Thus, FOXD1 resists cellular senescence and facilitates HNSCC cell proliferation by affecting the expression of p21/CDK2/Rb signaling, suggesting that FOXD1 may be a potential curative target for HNSCC.

## Introduction

Head and neck squamous carcinoma (HNSCC) is one of the most prevalent forms of cancer worldwide with an incidence rate ranked eighth and mortality rate ranked 12th in all cancer types in 2020, and the number of HNSCC patients is ~840,000, which is expected to increase to 1 million by 2030 [1]. Despite the recent progress in varieties of emerging therapies such as immunotherapy and targeted therapies, the five-year survival rate of HNSCC patients is ~60%, which is far from satisfactory [2–4]. Thus, it is imperative to discover novel therapeutic strategies for HNSCC.

Forkhead box (FOX) proteins consist of a series of structurally conserved transcription factor superfamilies, which are typically characterized by a unique DNA-binding forkhead structural domain. FOX proteins participate in multiple important biochemical procedures, such as apoptosis, invasion, proliferation, migration, and longevity [5–7]. Many of FOX proteins have been confirmed to be related to many types of cancers [8]. Using bioinformatics analysis, we demonstrated that forkhead box D1 (FOXD1), an important FOX family gene, was highly expressed in HNSCC. FOXD1 is located on chromosome 5q12, and it plays a major role in transcriptional regulation in embryogenesis. Recent studies have proved that FOXD1 is associated with tumorigenesis and cancer progression, serving as a prognostic indicator and a prospective target in some sorts of cancers [9–13]. In our research, we demonstrated that FOXD1 was over expressed in HNSCC tissues and related to bad overall survival (OS) and disease-free survival (DFS) according to The Cancer Genome Atlas (TCGA) database. Notably, FOXD1 has been reported to be an important transcriptional factor that promotes drug resistance and epithelial-to-mesenchymal transition (EMT) in oral carcinoma [14, 15]. However, the molecular mechanism of FOXD1 in HNSCC remains unclear.

Cellular senescence is defined as cell growth arrest characterized by a number of phenotypic alterations such as chromatin remodeling, metabolic reprogramming, cell morphological variations and upward adjustment of senescence-associated β-galactosidase (SA-β-gal) activity [16, 17]. A variety of biochemical factors such as oxidative stress, DNA damage or mitochondria injury may induced cellular senescence [18]. Many studies have elucidated that inhibition of cellular senescence accelerates and induction of senescence decelerates the cancer progression [19]. Thus, inducing senescence may be a potentially effective method for cancer therapy. An important molecular regulator of cellular senescence is the cyclin-dependent kinase (CDK) depressant, p21 (also called CDKN1A) [20].p21 promotes senescence of cancer cells through various pathways [21]. The p16INK4a/retinoblastoma (Rb) and p53/p21 pathways are the most important pathways involved in the induction and maintenance of senescence [22]. It has been reported that the cell senescence state indicates that cancer cells enter or reenter into irreversible cell cycle stagnation to decrease cell proliferation [22, 23]. A study proved that knockdown of FOXD1 facilitated the senescence of human mesenchymal stem cells and that intra-articular injection of a lentiviral vector encoding FOXD1 reduces the development of mouse osteoarthritis [24]. However, it remains unknown whether FOXD1 regulates the senescence of cancer cells. Therefore, we hypothesized that FOXD1 affects HNSCC cell senescence. As expected, the present findings suggested that FOXD1 plays a major role in mediating senescence by suppressing p21 in HNSCC, thus providing novel therapeutic strategies for cancer treatment.

Our research results showed that FOXD1 was post-transcriptionally regulated by microRNA miR-30e-5p, which inhibited its translation. In addition, we found that FOXD1 directly bound to the promoter of p21 and inhibited its transcription, which blocked the CDK2/Rb signaling pathway, thereby preventing the senescence of tumor cells and accelerating tumor cell proliferation. Thus, the present findings demonstrated the role and mechanism of FOXD1 in HNSCC cell senescence, suggesting that FOXD1 is a promising therapeutic target for HNSCC.

## Results

### High expression of FOXD1 is related to poor OS in HNSCC

The FOX family gene expression matrix was utilized to explore the role of FOX family genes in HNSCC by analyzing TCGA datasets. We discovered that most FOX family genes were highly expressed in tumors compared to normal tissues (Fig. S1A). Among them, FOXD1, FOXD2, FOXM1, FOXS1, FOXL1, FOXL2, and FOXI3 were dramatically overexpressed in HNSCC tissues compared to normal tissues [log fold change (FC) > 2] (Fig. S1B). Survival analysis indicated that FOXD1, FOXI3, FOXL1, and FOXL2 significantly affected the OS of patients (*p* < 0.05) (Fig. S1C). Above results were verified by qRT-PCR in six pairs of fresh specimens from HNSCC patients (Fig. S1D). There was no difference in the FOXI3, FOXL1, and FOXL2 mRNA levels between the tumor and normal tissues (Fig. S1E, F, G). Together, these findings indicated that high FOXD1 expression is related to poor OS in HNSCC patients.

### FOXD1 could be a promising prognostic indicator for HNSCC

**Fig. 1:**
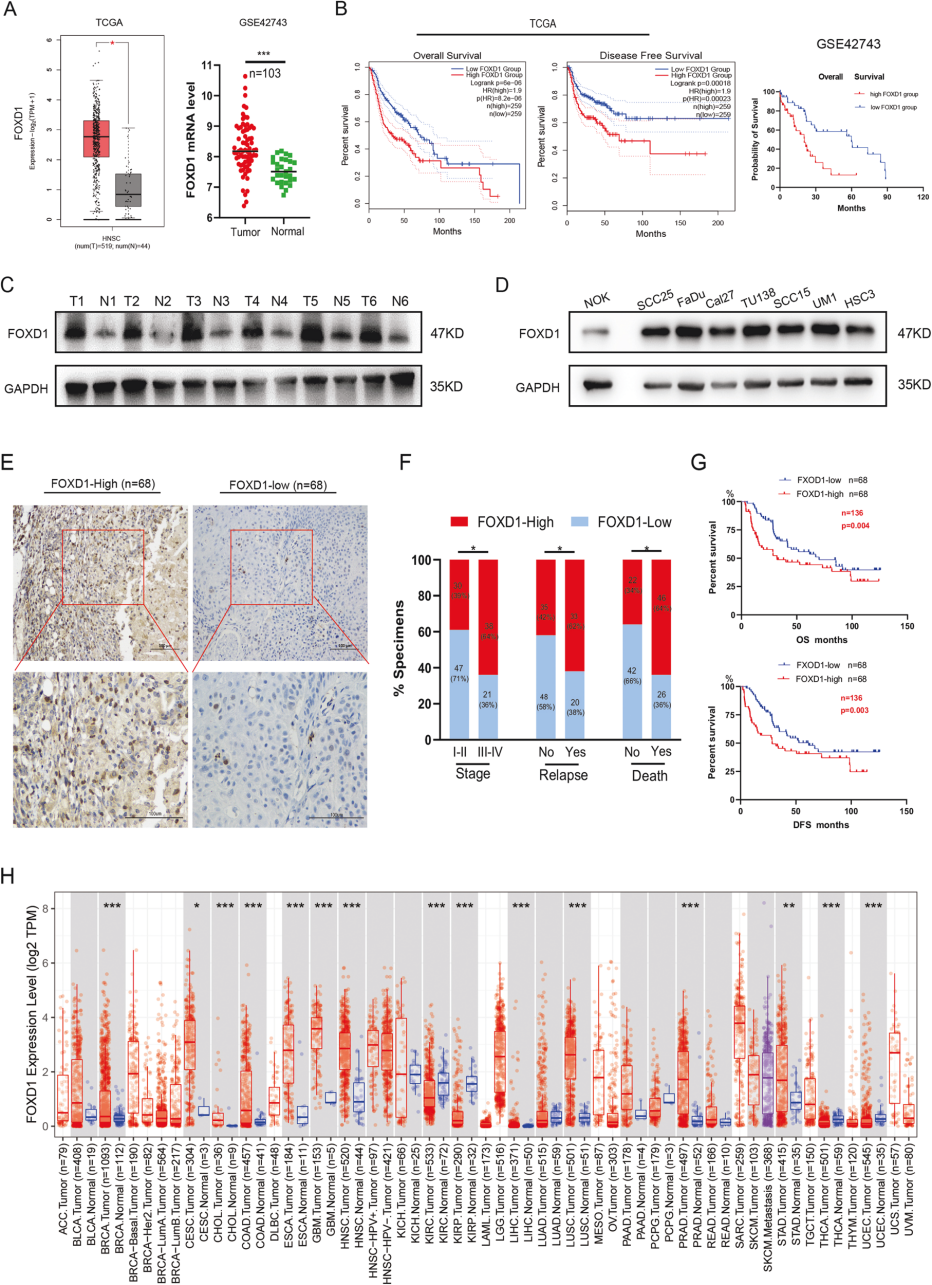
High expression of FOXD1 is associated with poor prognosis in patients with HNSCC. **A** Expression of FOXD1 in HNSCC and normal tissues in GSEA database. **B** Survival curves of high or low expression of FOXD1 in HNSCC in TCGA. **C** Western blot results of FOXD1 protein expression in HNSCC tissues (T) and corresponding adjacent normal tissues (N) in six patients with HNSCC. GAPDH was used as the loading control. **D** Western blotting. analysis of FOXD1 protein in normal oral epithelial cell line (NOK) and seven HNSCC cell lines (SCC25, FaDu, Cal27, TU138, UM1, HSC3, and SCC15). **E** Representative graphics of FOXD1 IHC staining in HNSCC paraffin sections. The samples were divided into FOXD1-high and FOXD1-low group based on their staining intensity. **F** Correlation analysis of FOXD1 expression level with various clinical pathological parameters of HNSCC patients (χ^2^ test). **G** Kaplan–Meier analysis of DFS and OS of patients with HNSCC stratified according to FOXD1 expression levels. **H** The expression level of FOXD1 in common types of cancers. The assays were repeated three times and all results are presented as the mean ± SEM. *p < 0.05, **p < 0.01, and ***p < 0.001. FOXD1 forkhead box D1, HNSCC head and neck squamous cell carcinoma, DFS disease-free survival, GSEA gene set enrichment analysis, GAPDH glyceraldehyde-3-phosphate dehydrogenase, IHC immunohistochemistry, OS overall survival, mRNA messenger RNA, TCGA The Cancer Genome Atlas, SEM standard error of the mean.

We next verified FOXD1 overexpression in HNSCC tissues (Fig. 1A), and we demonstrated that high FOXD1 expression corresponded to poor OS and DFS (Fig. 1B). Furthermore, western blot results implied that the FOXD1 protein level in tumors was also remarkably higher than that in adjacent normal tissues (Fig. 1C). Moreover, the FOXD1 protein expression in seven different HNSCC cell lines (SCC25, FaDu, Cal27, TU138, SCC15, UM1, and HSC3) was significantly higher than that in normal oral epithelium (NOK) cells (Fig. 1D). To further explore the clinical significance of FOXD1, we performed IHC on 136 HNSCC paraffin-embedded sections collected from our center, which demonstrated that high FOXD1 expression corresponded to worse clinical outcomes (Table S3). In addition, univariate and multivariate logistic regression analyses indicated that high FOXD1 expression could independently affect OS and DFS (Table S4–S5). Based on staining score, the samples were defined as FOXD1-high (68 cases) and FOXD1-low (68 cases) (Fig. 1E), and correlation analysis demonstrated that higher FOXD1 expression was associated with higher clinical stage, recurrence ratio, and death percentage (Fig. 1F). Furthermore, the Kaplan-Meier survival curve implied that the 5-year DFS and OS in FOXD1-high groups were worse compared to FOXD1-low groups (Fig. 1G). To better understand the role of FOXD1 in cancer, a pan-cancer analysis was performed. The FOXD1 expression matrix demonstrated that FOXD1 was over-expressed in various types of cancers (Fig. 1H) and corresponded to a bad survival (Fig. S2). Together, these findings suggest that FOXD1 is an prevailing oncogene and is related to the clinical stage of HNSCC, demonstrating that it could be a promising prognostic indicator for HNSCC.

### FOXD1 knockdown promotes the senescence and apoptosis but inhibits the growth of HNSCC cells

**Fig. 2:**
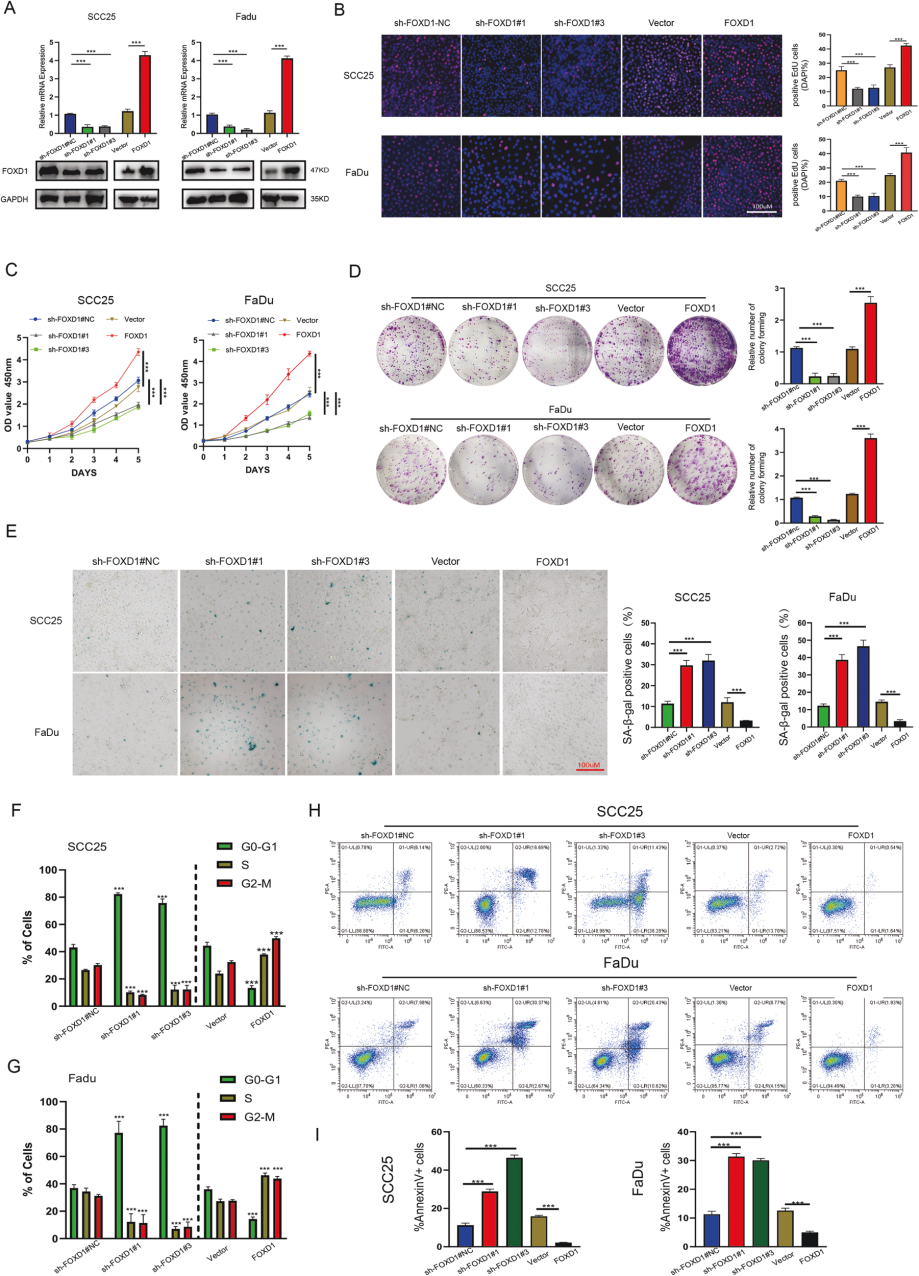
FOXD1 knockdown promotes the senescence and apoptosis but inhibits the growth of HNSCC cells, whereas FOXD1 overexpression results in the opposite trend. **A** The establishment of two HNSCC cell lines with knockdown and overexpression of FOXD1 was verified at the mRNA and protein levels. The (**B**) EdU, (**C**) CCK-8, and (**D**) colony formation assays showed that higher FOXD1 expression levels resulted in stronger proliferation abilities. **E** SA-β-gal staining showed that lower FOXD1 expression levels resulted in higher proportions of senescent tumor cells. **F**, **G** Cell cycle experiments showed that FOXD1 knockdown significantly increased the G0/G1 phase ratios and reduced the S phase ratios but that FOXD1 overexpression showed the opposite trend. **H**, **I** Apoptosis experiments showed that FOXD1 knockdown increased the percentage of apoptosis cells but that FOXD1 overexpression decreased the percentage of apoptosis cells. *p < 0.05, **p < 0.01, and ***p < 0.001. FOXD1 forkhead box D1, HNSCC head and neck squamous cell carcinoma, mRNA messenger RNA, EdU 5-Ethynyl-2’-deoxyuridine, SA-β-gal senescence-associated beta-galactosidase, SEM standard error of the mean.

In order to explore the oncogenic functions of FOXD1 in HNSCC, the SCC25 and FaDu HNSCC cell lines were used for several functional experiments. Firstly, FOXD1 was stably knocked down and overexpressed in these cell lines as verified at the mRNA and protein levels (Fig. 2A). The EdU, CCK-8, and colony formation assays revealed that the expression of FOXD1 was bound up with the proliferation ability of HNSCC. Knockdown of FOXD1 dramatically decreased the growth rate of HNSCC cells, while FOXD1 overexpression obtained the opposite results (Fig. 2B–D). Moreover, the number of senescent cells was the highest in sh-FOXD1 cells but the lowest in the FOXD1-overexpressing cells (Fig. 2E), which indicated that FOXD1 inhibited the senescence of HNSCC cells. To further understand this phenomenon, cell cycle and apoptosis experiments were conducted. As expected, FOXD1 knockdown significantly increased the G0/G1 phase ratios and reduced the S phase ratios, whereas FOXD1 overexpression showed the opposite trend (Fig. 2F, G). FOXD1 knockdown also increased the percentage of apoptosis cells, while FOXD1 overexpression significantly decreased the percentage of apoptosis cells (Fig. 2H, I). Together, these findings demonstrated that FOXD1 knockdown promotes the senescence and apoptosis but inhibits the growth of HNSCC cells.

### FOXD1 directly binds to the p21 promoter region and downregulates its expression

**Fig. 3:**
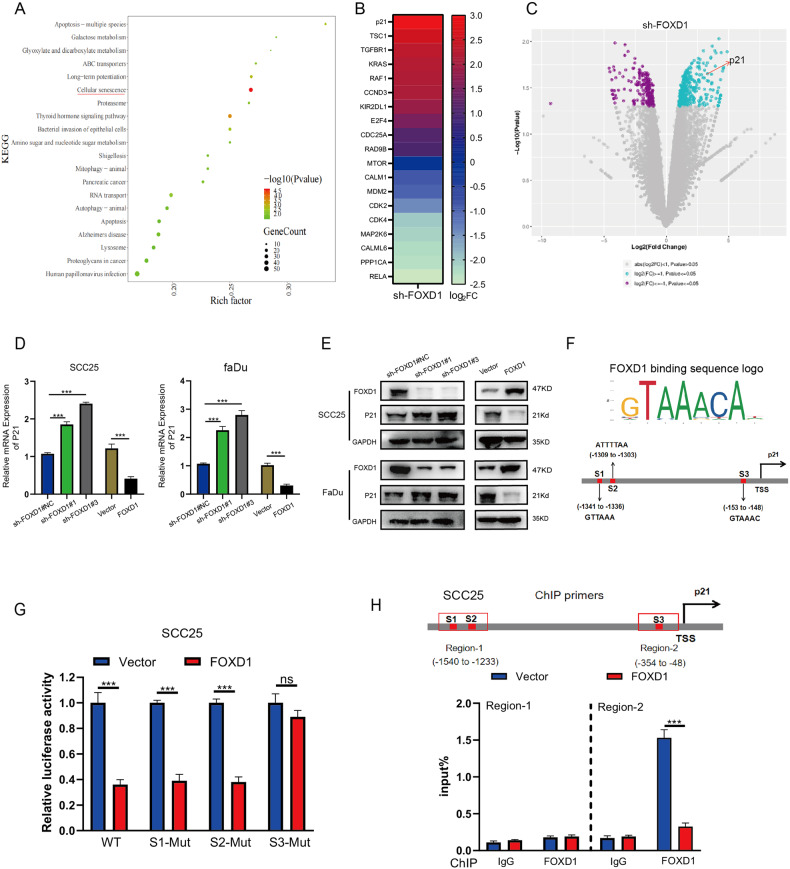
FOXD1 directly combined with the p21 promoter regions and downregulates its expression. **A** KEGG enrichment analyses between sh-FOXD1#NC and sh-FOXD1#1 SCC25 cells indicated enrichment in tumor progression. **B** Genes involved in the cellular senescence pathway are shown in the heatmap. **C** Volcano plot analysis indicated that p21 was notably upregulated in sh-FOXD1#1 SCC25 cells. **D** qRT-PCR and **E** western blot analyses clarified that p21 expression was remarkably increased in FOXD1 knockdown cells but significantly decreased in FOXD1-overexpressing cells. **F** Three regions (S1–S3) of the p21 promoter containing FOXD1-binding sites were predicted by JASPAR. **G** Luciferase assays confirmed that the relative luciferase activity was increased when S3 region was mutated. **H** ChIP-qPCR assay revealed the enrichment of FOXD1 on the p21 promoter (region 2) was significantly downregulated in FOXD1-overexpressed SCC25 cells (IgG was used as a control). *p < 0.05, **p < 0.01, and ***p < 0.001. FOXD1, forkhead box D1; KEGG, Kyoto Encyclopedia of Genes and Genomes; SEM standard error of the mean, ns negative significance, qRT-PCR quantitative real-time polymerase chain reaction, ChIP-qPCR chromatin immunoprecipitation-quantitative polymerase chain reaction.

To seek the mechanism of FOXD1 regulating the proliferation, senescence, and apoptosis of HNSCC cells, we used sh-FOXD1 and sh-FOXD1#NC (negative control) for RNA-seq analysis. KEGG analysis using the sequencing data indicated that the senescence pathway was enriched (Fig. 3A), which was consistent with the previous experimental results. To identify potential downstream genes, a matrix was generated according to the fold-change of multiple of genes enriched in the senescence pathway and proliferation pathway, which indicated that the fold-change of p21 (also known as CDKN1A) had the largest change (Fig. 3B). Volcano plot analysis of p21 indicated that the increase of p21 was high (Fig. 3C), which suggested that p21 may be the direct target gene of FOXD1. We next performed qRT-PCR and western blot analyses, which demonstrated that p21 expression was significantly increased in FOXD1 knockdown cells but remarkably decreased in FOXD1-overexpressing cells (Fig. 3D, E). JASPAR predicted that three regions (S1-S3) of the p21 promoter contain FOXD1-binding sites (Fig. 3F). After we mutated site S1, S2, S3 into Mut1, Mut2, Mut3 correspondingly, a dual-luciferase reporter assay was perpetrated to verify these potential FOXD1-binding sites in p21. We found when the S3 region was mutated, the relative luciferase activity was increased (Fig. 3G), which suggested that FOXD1 may bind to the S3 region of the p21 promoter. Besides, ChIP-qPCR assays indicated that FOXD1 can combine with the S3 region but not the S1 or S2 region at the promoter of p21 (Fig. 3H). Based on the above experimental results, we can confirm that the FOXD1 may combine with the p21 promoter via the S3 region and suppress its transcription.

### FOXD1 affects the proliferation, senescence, and apoptosis of HNSCC cells by regulating p21 protein expression

**Fig. 4:**
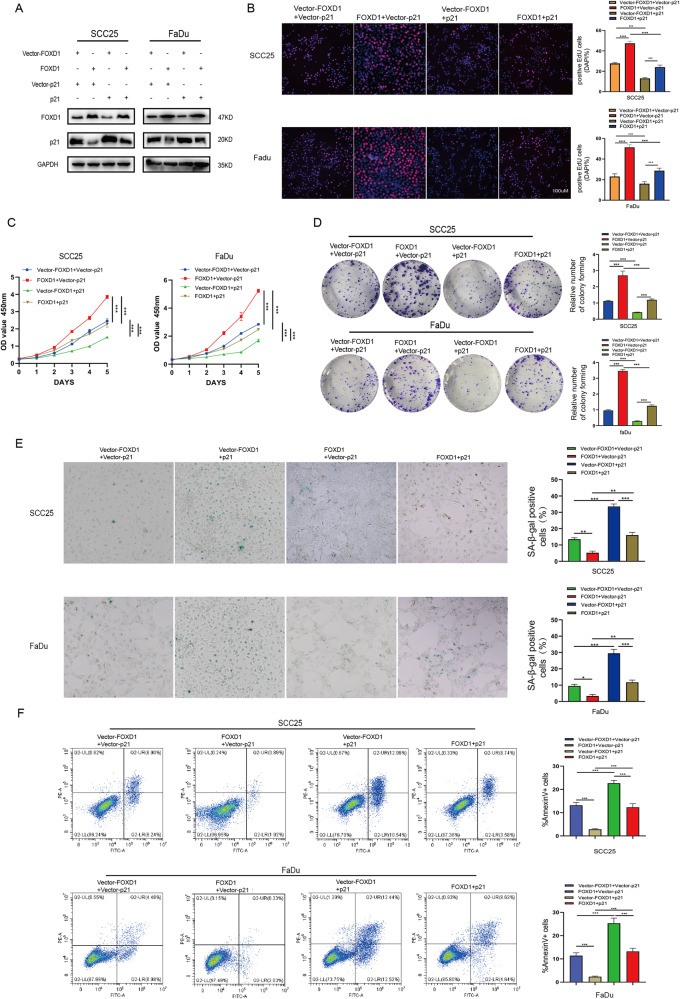
FOXD1 affects the proliferation, senescence, and apoptosis of HNSCC cells by regulating the level of p21 protein. **A** Western blot analysis verified the successful establishment of stable strains with simultaneous overexpression of FOXD1 and p21. The (**B**) EdU, (**C**) CCK-8, and (**D**) colony formation experiments revealed that p21 overexpression decreased cancer cell proliferation ability in SCC25 and FaDu cells. **E** SA-β-gal staining and (**F**) apoptosis assays showed that p21 overexpression increased the proportions of senescent and apoptotic HNSCC cells. FOXD1 forkhead box D1, HNSCC head and neck squamous cell carcinoma, EdU 5-Ethynyl-2’-deoxyuridine, SA-β-gal senescence-associated beta-galactosidase, SEM standard error of the mean.

Because FOXD1 negatively regulated the expression of p21, we overexpressed p21 in FOXD1-overexpressing SCC25 and FaDu cells for a rescue experiment to determine whether p21 inhibition is required for FOXD1-mediated promotion of HNSCC cell proliferation. Western blot analysis verified the successful establishment of stable cell lines with simultaneous overexpression of FOXD1 and p21 (Fig. 4A). Overexpression of p21 decreased the proliferative ability of cancer cells (Fig. 4B–D) as indicated by EdU, CCK-8, and colony formation assays in SCC25 and FaDu cells. Moreover, p21 overexpression increased the proportions of senescent and apoptotic HNSCC cells (Fig. 4E, F). Taken together, these findings demonstrated that FOXD1 affects the proliferation, senescence, and apoptosis of HNSCC cells by regulating the level of p21 protein.

### FOXD1 regulates the proliferation ability of HNSCC by affecting the activity of the p21/CDK2/Rb pathway without disturbing CDK4/6

**Fig. 5:**
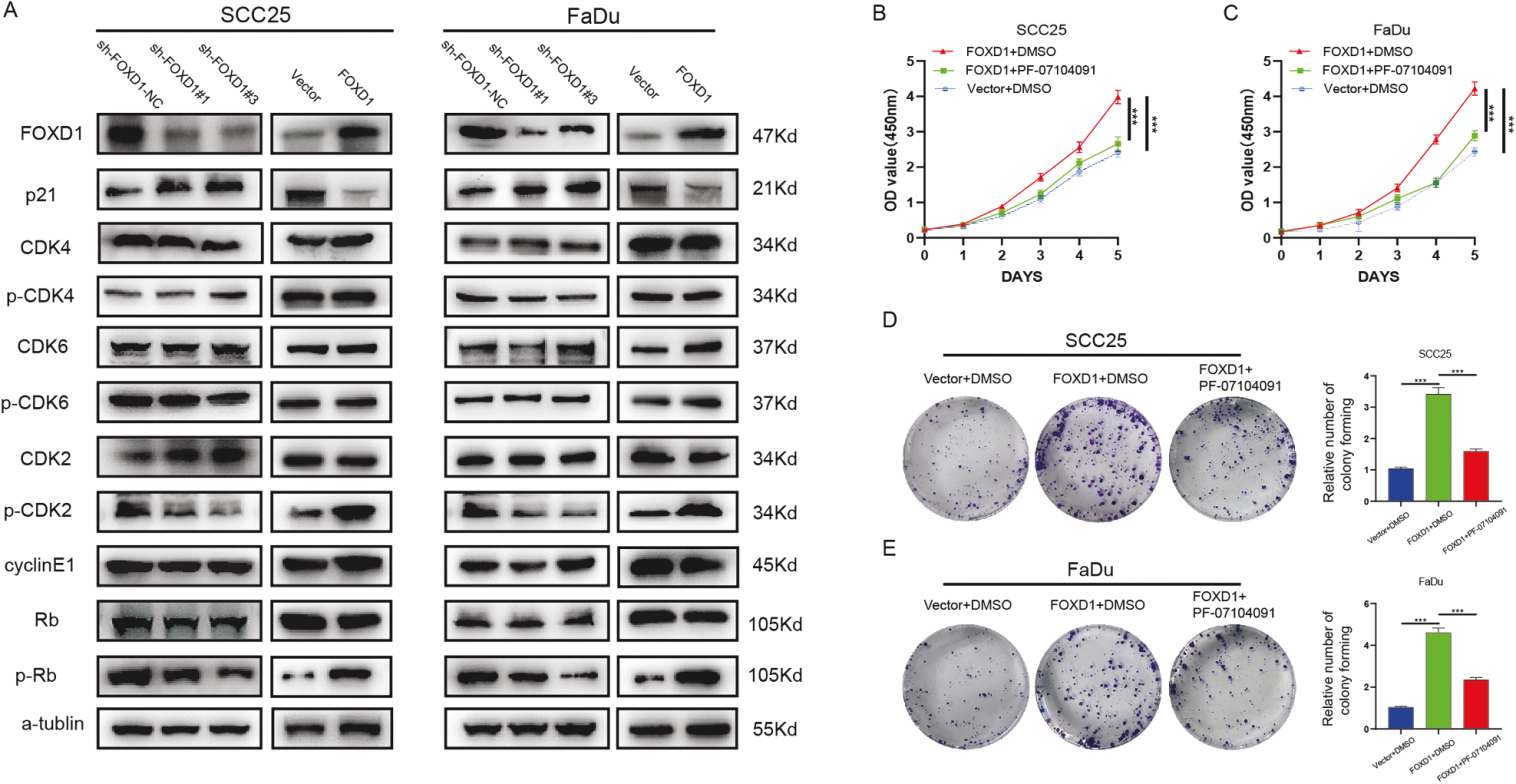
FOXD1 regulates the proliferation ability of HNSCC by affecting activity of the p21/CDK2/Rb signaling pathway without disturbing CDK4/6. **A** Western blot results showed that FOXD1 knockdown decreased the phosphorylation protein levels of CDK2 and Rb but that FOXD1 overexpression increased the phosphorylation levels of CDK2 and Rb without affecting the total protein levels. The CCK-8 assay (**B**, **C**) and colony formation assay (**D**, **E**) implied that the growth of FOXD1-overexpressing HNSCC cells was decreased after treatment with PF-07104091, a CDK2-specific inhibitor. *p < 0.05, **p < 0.01, and ***p < 0.001. FOXD1 forkhead box D1, HNSCC head and neck squamous cell carcinoma, CDK2 cyclin-dependent kinase 2, Rb retinoblastoma.

To further explore the molecular mechanism of FOXD1 regulating HNSCC cell proliferation, we detected the levels of molecules downstream of p21, including CDK2, CDK4, CDK6, cleaved caspase, cyclin E1, and Rb. Western blot analysis revealed that FOXD1 knockdown inhibited phosphorylated CDK2 and Rb but that FOXD1 overexpression increased the phosphorylation of these proteins without affecting their total protein levels. However, the expression level of FOXD1 did not affect the phosphorylated or total protein levels of CDK4, CDK6, cleaved caspase, or cyclin E1 (Fig. 5A). To explore the role of CDK2 in HNSCC, PF-07104091, a specific CDK2 inhibitor, was used to detect whether CDK2 acts as an anti-tumor role in HNSCC. As expected, the proliferation ability of the FOXD1-overepxressing HNSCC cells decreased after treatment with PF-07104091 (Fig. 5B–E). Together, these results suggested that the FOXD1/p21/CDK2/Rb axis has an important impact on the proliferation of HNSCC and that the CDK2 inhibitor reverses the malignancy of FOXD1-overexpressing HNSCC cells to a certain extent.

### Knockdown of FOXD1 inhibits tumor growth in vivo

**Fig. 6:**
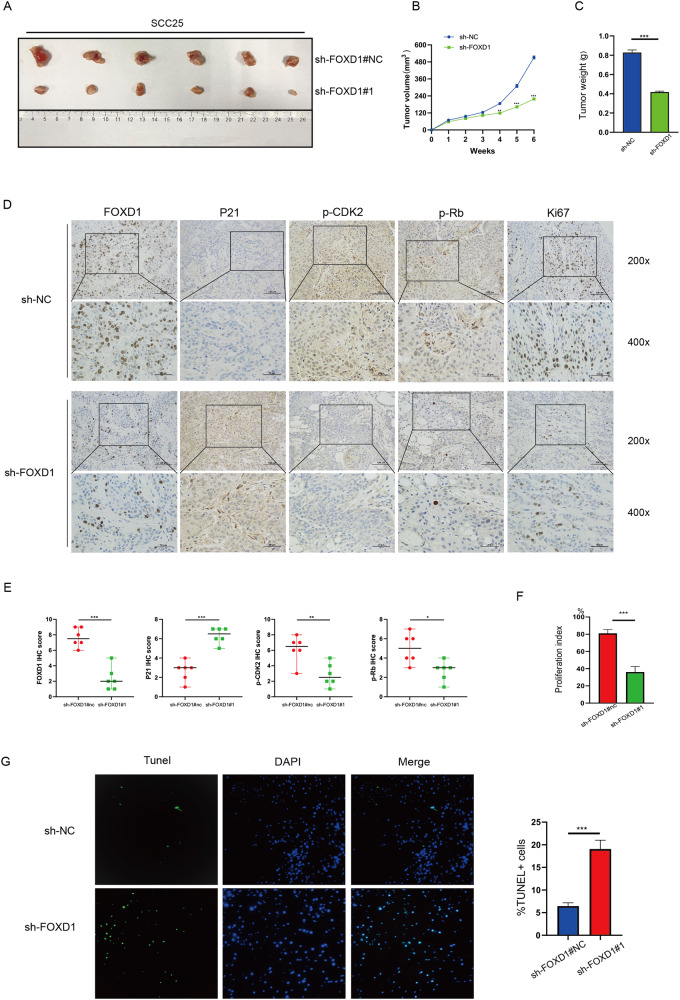
Knockdown of FOXD1 suppresses the growth of tumors in vivo. **A** Tumors in group sh-FOXD1#1 were notably smaller than those in group sh-FOXD1#NC. **B** Tumor volumes in the FOXD1 sh-NC and sh-FOXD1 groups. **C** Tumor weights in the FOXD1 sh-NC and sh-FOXD1 groups. **D** IHC was performed on the mouse tumor tissues to evaluate the expression of FOXD1, p21, p-CDK2, p-Rb, and Ki67 (a proliferation marker). Immunohistochemistry score (**E**) and proliferation index (**F**) of the expression levels of FOXD1, p21 p-CDK2, p-Rb, and Ki67. **G** TUNEL assay indicated that the sh-FOXD1 group had more apoptotic cells than the sh-NC group. *p < 0.05, **p < 0.01, and ***p < 0.001. FOXD1 forkhead box D1, p-CDK2 phosphorylated cyclin-dependent kinase 2, p-Rb phosphorylated retinoblastoma, TUNEL terminal deoxynucleotidyl transferase dUTP nick end labeling, SEM standard error of the mean.

To further research the role of FOXD1 in vivo, we injected SCC25 cells with different FOXD1 expression levels (FOXD1-sh#NC and FOXD1-sh#1) subcutaneously into nude mice. At 6 weeks postinjection, the nude mice in each group were executed, and the tumors were gathered and weighed. Compared to the sh-FOXD1#NC (negative control) group, the tumors in the sh-FOXD1#1 group were notably smaller (Fig. 6A–C). The tumors were also evaluated by IHC to detect the protein level of FOXD1, p21, p-CDK2, p-Rb, and Ki67 (a proliferation marker) (Fig. 6D). According to the immunohistochemical score and proliferation index, FOXD1, p-CDK2, p-Rb, and Ki67 were downregulated, while p21 was upregulated in FOXD1 knockdown mouse tumors (Fig. 6E, F). In addition, the TUNEL assay results demonstrated that there were more apoptotic cells in the sh-FOXD1#1 group (Fig. 6G). As we expected, the in vivo results were consistent with the in vitro experiments. Collectively, these data suggested that FOXD1 may promote tumor malignancy in HNSCC by inducing the p21/CDK2/Rb pathway.

### miR-30e-5p inhibits HNSCC cell proliferation by targeting and repressing FOXD1 expression in vitro and in vivo

**Fig. 7:**
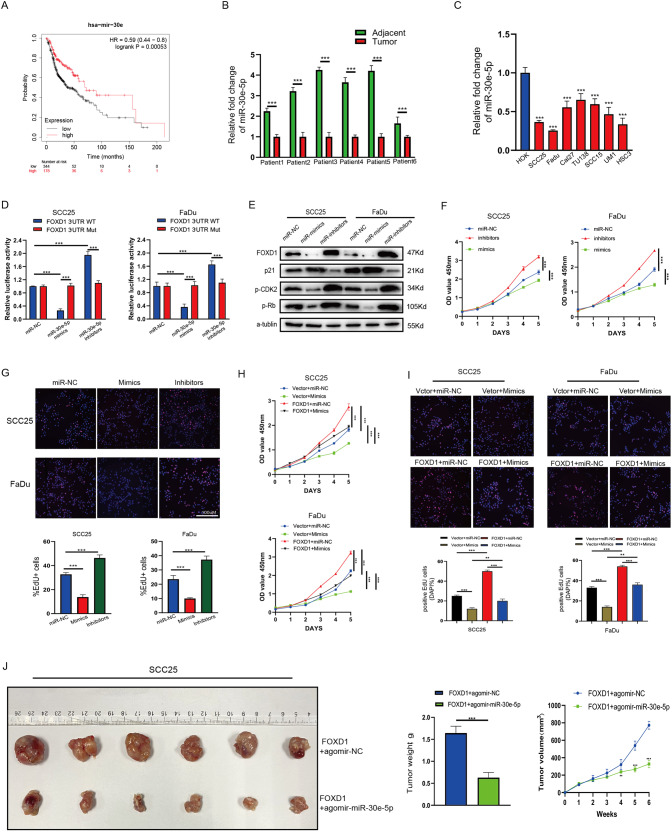
miR-30e-5p inhibits cell proliferation by targeting and repressing FOXD1 expression in vitro and in vivo in HNSCC. **A** Kaplan–Meier plot curves implied that down-expression of miR-30e was related to bad OS in HNSCC. **B** qRT-PCR result revealed that miR-30e-5p was remarkably downregulated in tumor HNSCC tissues by comparison with corresponding adjacent normal tissues. **C** miR-30e-5p was dramatically downregulated in HNSCC cells according to qRT-PCR analysis. **D** Dual-luciferase reporter assays indicated that miR-30e-5p directly bound to the 3’-UTR of FOXD1. (E) FOXD1, p21, p-CDK2, and p-Rb protein levels in cells transfected with miR-138-5p mimics or inhibitor. **F**, **G** Compared to miR-control cells, the proliferative capacity of cells was significantly suppressed after transfection of miR-138-5p mimics but promoted after transfection of miR-138-5p inhibitors. **H**, **I** The proliferative capacity of cells was reversed after transfection of miR-30e-5p mimics into FOXD1-overexpressing cells. **J** miR-30e-5p overexpression dramatically restrained the proliferation of HNSCC cells in vivo. *p < 0.05, **p < 0.01, and ***p < 0.001. HNSCC head and neck squamous cell carcinoma, FOXD1 forkhead box D1, OS overall survival, qRT-PCR quantitative real-time polymerase chain reaction, 3’-UTR 3’-untranslated region.

To further investigate the upstream microRNA of FOXD1, we used TargetScan (targetscan.org) to predict miRNAs that may target the FOXD1 3’-UTR, which identified eight potential miRNAs (Table S6). The survival curves of the eight miRNAs in HNSCC were generated using the Kaplan-Meier plotter databases. The results implied that high expression of miR-30e (Fig. 7A), mir-30a, and mir-216b (Fig. S3A) was significantly related to good OS in HNSCC, while the remaining five miRNAs showed no significant correlation with OS (Fig. S3B). qRT-PCR assay results showed that only miR-30e-5p was significantly downregulated in tumor tissues (Fig. 7B), while there was no difference in miR-30a-5p and miR-216b-5p expression between the tumor and normal tissues (Fig. S3C). We further detected the expression of miR-30e-5p in HNSCC cell lines and normal cell lines, which indicated that miR-30e-5p was notably decreased in cancer cells (Fig. 7C). Furthermore, the upregulated miR-30e-5p predicted better prognosis in some types of cancers according to the Kaplan-Meir plotter databases (Fig. S3D).

**Fig. 8:**
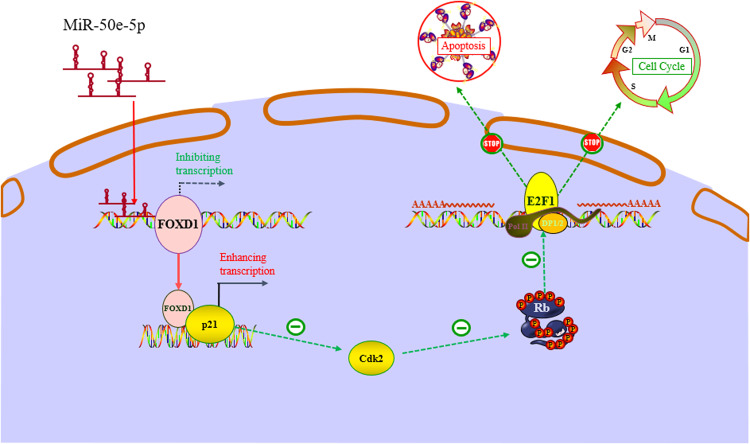
Mechanism diagram of this study. MiR-3oe-5p acted as a tumor suppressor by repressing the translation of FOXD1 post-transcriptionally through binding to the 3’-untranslated region (UTR). The downregulation of FOXD1 results in diminished transcriptional regulation of p21, leading to an increase in p21 expression levels. Consequently, the inhibitory effect on CDK2 is strengthened. Moreover, the decreased expression of CDK2 reduces the phosphorylation level of Rb protein, thereby inhibiting the cell cycle and promoting cellular senescence and apoptosis.

Dual-luciferase reporter assay indicated that miR-30e-5p directly bound to the 3’-UTR of FOXD1 (Fig. 7D). Western blot analysis prompted that miR-30e-5p mimics downregulated the expression of FOXD1, p-CDK2, and p-Rb but upregulated the expression of p21, while miR-30e-5p inhibitors caused the opposite effects (Fig. 7E). The CCK-8 and EdU assays demonstrated that transfection of miR-30e-5p mimics reduced HNSCC cell proliferation, but that transfection of miR-30e-5p inhibitors increased HNSCC cell proliferation (Fig. 7F, G). To further verify that miR-30e-5p affects the proliferation of HNSCC cells by regulating the expression of FOXD1, miR-30e-5p mimics, and inhibitors were transfected into FOXD1-overexpressing cells and corresponding control cells. These rescue experiments demonstrated that miR-30e-5p mimics inverted the promoting effect of FOXD1 overexpression on cell proliferation in vitro and in vivo (Fig. 7H, J). To sum up, these results demonstrated that miR-30e-5p inhibits the translation of FOXD1 and regulates the proliferation of HNSCC via the FOXD1/p21/CDK2/Rb signaling pathway. Mechanism diagram of this study was shown in Fig. 8.

## Discussion

FOX proteins comprise a superfamily of evolutionarily conserved transcription factors characterized by a distinct DNA-binding forkhead domain. FOXA1, FOXM1, FOXO subfamily genes, and FOXR1 have been widely studied and confirmed to promote tumor progression through various pathways, such as proliferation, invasion, and immunity [5–8]. Analysis of the data of HNSCC in TCGA demonstrated that FOXD1, FOXD2, FOXM1, FOXS1, FOXL1, FOXL2, and FOXI3 were markedly overexpressed in HNSCC tissues in contrast with normal tissues. Furthermore, survival analysis of these FOX genes revealed that FOXD1, FOXI3, FOXL1, and FOXL2 significantly affected the OS of HNSCC patients. However, qRT-PCR analysis using fresh specimens of HNSCC patients indicated that only FOXD1 mRNA expression was significantly upregulated in HNSCC tissues compared to corresponding adjacent normal tissues. Thus, these findings suggested that FOXD1 may be a prognostic factor for poor OS in HNSCC patients.

FOXD1 has been reported to promote tumor progression in many cancer types. FOXD1 upregulates vascular endothelial growth factor A (VEGFA), which accelerates tumor angiogenesis in colorectal cancer [25]. lncRNA CYTOR, which promotes EMT and chemoresistance, has been reported to be regulated by FOXD1 transcription in oral carcinoma [26]. Furthermore, FOXD1 can increase resistance to targeted drugs by transcriptionally activating the expression of connective tissue growth factors [27]. FOXD1 has been reported to act as a malignant factor in gastric intestinal metaplasia [28] and glioma [29]. In the present study, analysis of TCGA databases and in vitro experiments demonstrated that FOXD1 was overexpressed in HNSCC and that overexpressed FOXD1 was positively correlated with adverse clinical prognostic indicators. In addition, the present findings indicated that FOXD1 overexpression promoted, but FOXD1 knockdown inhibited HNSCC cell growth by affecting cell senescence and apoptosis. Collectively, these findings indicated that FOXD1 is a promising prognostic marker and molecular target for HNSCC.

We next investigated the molecular mechanism of FOXD1 in HNSCC. We performed a series of experiments in HNSCC cell lines and found that FOXD1 dramatically affect cellular senescence. FOXD1 knockdown significantly increased the proportions of senescent cells, proportions of apoptotic cells, and G0/G1 phase ratios, which decreased the proliferation ability of HNSCC cells, while FOXD1 overexpression resulted in the opposite trend. These results suggested that the FOXD1 transcription factor is upregulated in HNSCC and that it regulates the proliferation ability of HNSCC cells by affecting the cell cycle. In addition, the present study indicated for the first time that FOXD1 has vital impact on regulating the senescence of cancer cells. p21 is one of the most important cyclin-dependent kinase inhibitors (CDKIs), and it plays key roles in regulating the cell cycle and cellular senescence [30–33]. In our research, we proved that FOXD1 directly bound to the p21 promoter regions and downregulated its expression, thereby inhibiting the senescence of cancer cells. Thus, the present results in combination with previous literature suggest that FOXD1 is a factor that promotes tumor cell malignancy through various pathway. The role of FOXD1 in HNSCC has been mostly investigated with bioinformatics analyses, and basic research is lacking. The present study suggested that FOXD1 directly affects the senescence of HNSCC cells by regulating p21, which significantly affects HNSCC cell proliferation both in vitro *and* in vivo, thereby providing a new theoretical basis for understanding the molecular mechanism of HNSCC. Thus, these findings suggested that FOXD1 may be an important target for HNSCC. But we should realize that although cellular senescence seems like a good anti-tumor strategy, senescent cells can also activate the release of many molecules such as proinflammatory cytokines, chemokines and proteases, which gathered within the senescence-associated secretory phenotype (SASP). And SASP factors can paradoxically promote the growth and invasion of tumor cells [34–37]. In order to overcome this contradictory biological phenomenon, a recent research had found the therapeutic concept that chimeric antigen receptor (CAR) T cells targeting senescent cells can be effective senolytics [38]. Another study used a specific drug, XL413, to induce senescence in hepatocellular carcinoma cells, followed by another specific drug, AZD8055, to induce apoptosis in the senescent cells. The combination of these two drugs significantly reduced the proliferation of hepatocellular carcinoma cells and improved the survival rate of hepatocellular carcinoma mice [39]. It seems to be a very promising therapeutic strategy where we first induce senescence in cancer cells and then target and remove them. The next step of our research will focus on removing senescent cells.

CDK4/6 and CDK2 are the dominant CDKs that control cell G1/S transition though finely tuned regulation of the phosphorylation of the Rb cancer suppressor, which subsequently releases E2F, a transcriptional factor involved in cell cycle regulation [40, 41]. Because p21 plays a key role in the cell cycle by inhibiting CDKs, we further examined the downstream pathway of p21 by evaluating the total protein and phosphorylated levels of related downstream genes. The present results indicated that p21 inhibited the activity of the CDK2/Rb pathway without disturbing CDK4/6 in HNSCC, and a specific CDK2 inhibitor reduced the proliferation ability of FOXD1-overexpressing cells by affecting the activation status of this pathway. Recently, CDK4/6 inhibitors have been reported to show promising antitumor activity in HNSCC, and they have been demonstrated to be safe and effective in recurrent/metastatic HNSCC [42]. Although CDK2 inhibitors are difficult to develop due to the high selectivity of CDK2, CDK2 is an attractive target in treating tumors of specific genotypes [43]. The combination of CDK2 inhibitors and CDK4/6-inhibitors appears to be effective against cancer cells resistant to CDK4/6 inhibitors [44–47]. Recently, PF-07104091, a CDK2-specific inhibitor, has been reported to be effective and highly selective [48]. The present results preliminarily demonstrated the therapeutic effect of PF-07104091 on HNSCC cells, providing a theoretical basis for its application in HNSCC in the future.

miRNAs are small noncoding RNAs that act mainly on the 3’-UTR of target genes, thus regulating gene expression [49]. In the present research, we used bioinformatics analysis to screen out the potential miRNAs that regulate FOXD1, which identified miR-30e-5p. MiR‐30e‐5p has been reported to play key role in anti-tumor therapy, and it is downregulated in chronic myeloid leukemia [50], glioma [51], and breast cancer [52]. However, miR‐30e‐5p is upregulated in lung adenocarcinoma, contributing to tumor proliferation and invasion [53]. These studies suggest that miR‐30e‐5p may be a potential cancer therapeutic agents. Our study expounded that miR-30e-5p expression was downregulated in HNSCC tissues compared to corresponding adjacent normal tissues and that miR-30e-5p overexpression was associated with a better prognosis in HNSCC. Furthermore, miR-30e-5p overexpression promoted the senescence and apoptosis of HNSCC cells, which inhibited the growth of cells, whereas downregulation of miR-30e-5p had the opposite trend. More importantly, we demonstrated that miR-30e-5p bound to the 3’-UTR of FOXD1 and repressed FOXD1 translation post-transcriptionally, thus activating the p21/CDK2/Rb signaling pathway. miR-30e-5p serves as a tumor suppressor inhibiting the FOXD1-mediated progression of HNSCC.

## Conclusions

In summary, the present study demonstrated that FOXD1 mRNA and protein are overexpressed in HNSCC tissues compared to corresponding adjacent normal tissues and that high FOXD1 expression corresponds to poor prognosis. We also discovered that FOXD1 knockdown increases the percentage of senescent cells in HNSCC and decreases the proliferation ability of HNSCC cells. FOXD1 binds to the p21 promoter and inhibit its transcription, which inhibits the CDK2/Rb signaling pathway, thus preventing tumor cell senescence and accelerating tumor cell proliferation. Our mechanistic research suggested that miR-30e-5p is a tumor suppressor in HNSCC by post-transcriptionally repressing the translation of FOXD1 through binding to the 3’-UTR. As a result, FOXD1 resists cellular senescence and facilitates HNSCC cell proliferation by affecting the expression of the p21/CDK2/Rb signaling pathway, suggesting that blockade of the FOXD1/p21/CDK2/Rb axis may be a potential treatment for HNSCC.

## Materials and methods

### Cell culture

The SCC25, FaDu, Cal27, TU138, SCC15, UM1, and HSC3 HNSCC cell lines as well as the normal oral epithelial cell (NOK cell line) were obtained from iCell Bioscience Inc. An incubator containing 5% CO_2_ was used to incubate all cell lines at 37 °C in a humidified environment. Cell lines were confirmed by STR identification and routinely tested for mycoplasma free using Genechem’s MycoFreeTM mycoplasma detection kit.

### Patients and samples

Our research gathered 136 HNSCC paraffin-embedded samples from patients with histopathological and clinical diagnosis at Sun Yat-sen University Cancer Center (SYSUCC; from 2010 to 2020) after informed consent was obtained. Neither chemotherapy nor radiotherapy was administered before surgery, and all samples tested negative for HPV. The clinical pathological data were acquire from electronic medical records. We gathered 6 pairs of fresh HNSCC tissues from HNSCC patients diagnosed by pathology in our cancer center. The paired normal tissues were taken from 1 to 3 cm around the tumor focus.

### RNA isolation and quantitative real-time polymerase chain reaction (qRT-PCR)

RNA extraction was performed using a specific RNeasy Plant Mini Kit (ESscience, Cat. No. RN001) and quantified by a Nanodrop ND1000. Reverse transcription assay was utilized to generate complementary DNA and qRT-PCR was completed by RealStar Power SYBR qPCR Mix (GenStar, Cat. No. A311-01). The calculation of expression of related genes was performed by the 2^−ΔΔCT^ method. The relevant primer sequences are showed in Supplementary Table S1.

### Western blot analysis

Samples were lysed using specialized protein lysis solution (Beyotime, Cat. No. P0013B) supplemented with protease inhibitor cocktail (CWbio, Cat. No. CW2383S) for 30 min on ice. The lysed solution was centrifuged at 16,000 rpm at 4 °C for 25 min. The protein concentration was detected by a bicinchoninic acid (BCA) test kit (Beyotime, Cat. No. P0010). Protein samples (20 μg/well) were separated by a specific FuturePAGE™ Protein Precast Gel (ACE biotechnology, Cat. No. ET12010Gel) and then shifted to polyvinylidene fluoride (PVDF) membranes (Roche, Cat. No. 3010040001). Skim milk (5%) was used for blocking process. After that, we hatched the membranes for 12 h at 4 °C with the diluted primary antibodies. The membranes were then hatched with the diluted secondary antibodies for 1.5 h at 25 °C. All antibodies used in our study were listed in Supplementary Table S2.

### Constructs, transfection, and retroviral infection

FOXD1 and control short hairpin RNAs (shRNAs) were purchased from GeneCopoeia. The coding sequence of FOXD1 and p21 was amplified and cloned into vector pLVX-IRES-Puro. The recombinant lentiviral vectors were co-transfected with the packaging plasmids pSPAX2 and pMD2.G using Lipo8000™ (Beyotime Biotechnology, Cat. No. C0533) Transfection Reagent. Lentivirus infection of cancer cells were added with polybrene (Sigma-Aldrich, 8 μg/ml) for 12 h. Puromycin (0.5 g/mL) was used to screen stable cell lines. Two effective shRNAs targeting FOXD1 were designed as follows:

shRNA#1,GGATCCCCCGTATATCGCGCTCATCACTATTCAAGAGATAGTGATGAGCGCGATATACGTTTTTAAGCTT;

shRNA#3,GGATCCCCTTGTTAATAACGCTATGTTAGTTCAAGAGACTAACATAGCGTTATTAACAATTTTTAAGCTT.

### Colony formation and CCK-8 assays

Colony formation experiments were performed to explore the proliferative capability of HNSCC cells. Briefly, six-well plates were seeded with 1000 cells per well and cultured for about 2 weeks in complete medium. The number of cell colonies was counted after 14 days. CCK-8 assays were carried out according to the following steps. Firstly, appropriate number of cells (2000-5000 cells/well) were seeded into 96-well plates. For 5 consecutive days, CCK-8 solution (Dojindo, Cat. No.CK04) was diluted 1:10 with the medium and then added to the 96-well plates for 1 h. At last, a specific microplate reader was applied to determine absorbance at 450 nm.

### Chromatin immunoprecipitation (ChIP)-quantitative polymerase chain reaction (qPCR)

ChIP assays were conducted using a specific ChIP assay kit, SimpleChIP® Enzymatic Chromatin IP Kit (CST, Cat. No. 9003). In brief, cell samples were cross-linked by 1% formaldehyde for 12 min, lysed, and sonicated on ice to generate DNA fragments (ranging from 300 to 1200 bp). After that, the cell lysates were hatched with the specific ChIP-grade antibody for one night and then subsequently hatched with protein G-agarose beads for one day at 4 °C. We cleaned and eluted the beads, and make sure that the cross-links were inverted by incubation at 66 °C for 4.5 h. At last, purified DNA was performed to detect the combining of FOXD1 to the p21 promoter by PCR. The following ChIP-qPCR primers were used:

p21-ChIP-F1&2, AGGCCCACAAGGACTCTCATAG;

p21-ChIP-R1&2, AATCAAGGCATAAAAATTTCATTGT;

p21-ChIP-F3, TTTCTGGCCGTCAGGAACATG;

p21-ChIP-R3, CACAAGCACACATGCATCAGATC.

### Immunohistochemistry (IHC)

IHC staining of paraffin-embedded HNSCC tissue sections (*n* = 136) were performed using the corresponding antibodies. The detailed immunohistochemical scoring and grouping methods are described in our previous report [54]. For statistical analysis, we divided the samples into FOXD1-high group and FOXD1-low group according to the staining score we calculated.

### 5-Ethynyl-2’-deoxyuridine (EdU) assay

A specific EdU kit (Ribobio, Cat. No. C10310-1) were utilized to measure the proliferative capability. In brief, logarithmic growth stage cells were plated in 24-well plates at a suitable density. The EdU solution was diluted 1:1000 with culture medium, and the solution was then mixed with cells for 1.5 h. Cells were then fastened with 75% ethylalcohol for 25 min, washed several times with phosphate-buffered solution (PBS), and stained with Apollo staining reaction solution. After staining, Hoechst 33342 was used for staining cell nuclei for 25 min. Pictures were then obtained using a fluorescence microscope.

### Terminal deoxynucleotidyl transferase dUTP nick end labeling (TUNEL) assay

For detecting apoptosis situation in tissue samples, a TUNEL kit was utilized (Elabscience Biotechnology, Cat. No. E-CK-A320) following by the manufacturer’s instructions. First, tissue specimens were dewaxed and hydrated using standard procedures. A specific labeling solution was then well mixed followed by the manufacturer’s instructions, and 100 μL of TdT equilibration buffer was put into each slide followed by incubation for 10–30 min at 37 °C. The TdT equilibration buffer was then aspirated, and 50 μL of labeling buffer was mixed with each slide followed by incubation in a moist black box at 37 °C for 60 min. The redundant liquid was sucked out with blotting paper, and the slides were sealed with a sealer containing anti-fluorescence quencher. Finally, we obtained images by operating a fluorescence microscope with FITC.

### Cell cycle analysis

A specific cell cycle assay kit (KeyGEN bioTECH, Cat. No. KGA512) was performed for cell cycle analysis. In summary, cells were digested with ethylenediaminetetraacetic acid (EDTA)-free trypsin, washed several times with PBS, and fastened in 75% ethylalcohol at −18 °C for one night. After that, the fixed cells were washed several times with ice-cold PBS and hatched with propidium iodide (PI) at 25 °C in a wet black box for half an hour. Flow cytometry (ACEA NovoCyte flow cytometer) was used for cell cycle distribution analysis.

### Apoptosis assay

To detect apoptotic cells, a specific Apoptosis Detection Kit (Beyotime Biotechnology, Cat. No. C1062M) was utilized following by the manufacturer’s instructions. Cell samples were digested with trypsin, washed three times with pre-chilled PBS, and then counted. Cells (100,000 cells per tube) were stained with 6 μL of Annexin V-FITC and 12 μL of PI for half of 1 h in a dark kit. Samples were then detected via flow cytometry (cytoFLEX cytometer).

### Senescence-associated β-galactosidase (SA-β-gal) staining

A specific *SA-β-gal* staining kit (Beyotime Biotechnology, Cat. No. C0602) was utilized following by the manufacturer’s instruction for detecting cell senescence. Firstly, we rinsed the cells with PBS three times after sucking out the medium. Then, 600 μl β-gal staining solution was put into the cells followed by incubation at 25 °C for 20 min. The staining working solution was then prepared and added to the fixed cells followed by overnight incubation at 37 °C without CO_2_. Photos were then obtained using a fluorescence microscope.

### Xenograft tumor model

Cancer cells in a logarithmic growth phase were collected, digested with trypsin, washed with PBS, and resuspended in PBS. After randomly dividing the 4–6-week-old BALB/c male nude mice into control (*n* = 6) and treatment groups (*n* = 6), the mice were subcutaneously injected with cells (1 × 10^6^ cells per mouse). Subcutaneous tumors started to form at one week postinjection, and the tumor volumes were calculated every 4 days. After 28 days, all of mice were put to death . The tumors were collected, weighed, and embedded in paraffin, and sectioned for subsequent experiments.

### Dual-luciferase reporter assay

Firstly, cells (2 × 10^5^) were seeded into a 6-well plates for one day. The next day, 100 ng of luciferase reporter plasmid with wild-type or point mutated binding site was transfected into cell lines (pSPAX2 and pMD2.G were used for packaging plasmids). The specific transfection process was the same as Method section “Constructs, transfection, and retroviral infection”. Dual-luciferase reporter assay was performed by a specific Luciferase assay Kit (Beyotime technology, Cat. No. RG027). Relative firefly luciferase activity was standardized to Renilla luciferase activity as a control for transfection efficiency. The three cloned primer sequences are as follows:

p21-pro-F GCTAGCCCGGGCTCGAGTGACGAGCCCTCAGTCTTCTTG

p21-pro-R ACCGGAATGCCAAGCTTAGATCCCAGCCCTGTCGCAAG

p21-mut1-R1 CAAAAACCTAAATTGTTCAACTGTCCAATTTAAGATAG

p21-mut1-F2 GTTGAACAATTTAGGTTTTTGAATGAATGGATG

p21-mut2-R1 TAAATTGCCTCCTAATTTTAATTTTCATCCATTCATTCAAAAAC

p21-mut2-F2 TAAAATTAGGAGGCAATTTATATTTAAAAATG

p21-mut3-R1 CTGGGGTCTTTAGAGGTCTC

p21-mut3-F2 GAGACCTCTAAAGACCCCAGCATTTGCTTAGCCTGTTACTCTGAACAG

### RNA-sequencing (RNA-seq)

The RNA extraction process was the same as Method section “RNA isolation and quantitative real-time polymerase chain reaction (qRT-PCR).” RNA quality testing assay was performed using electrophoresis (Invitrogen, Cat. No. 10488096). Samples with RNA integrity number (RIN) values of >9 were considered to be included in the further analysis. RNA-seq libraries were completed by Majorbio Corporation (Shanghai, China).

### Bioinformatics analysis

All mRNA sequencing data were downloaded from TCGA data portal. GEO accession number GSE42743 which was conducted by Holsinger C et al. from Stanford University School of Medicine was used to explore differences of gene expression between tumors and corresponding normal tissues in HNSCC. The relevant survival analysis and expression data of relevant genes were processed by a custom C++ program. TIMER 2.0, GEPIA2, and KM-plotter were used for pan-cancer analysis of FOXD1.

### Statistical analysis

Statistical analyses were performed using SPSS software version 26.0 (IBM Corp., Armonk, NY, USA) and GraphPad Prism9. Statistical methods mainly included chi squared test (χ^2^ test), Spearman’s rank correlation analysis, and student’s *t*-test (two tailed). Multivariate Cox expression analysis was performed to seek the relevance between gene expression and assorted clinicopathological factors. *p* < 0.05 was considered statistically significant.

### Supplementary information

Supplementary Materials-Figures

Supplementary Materials-Tables

Original Data File

## Data Availability

The original western blots are shown in Supplementary Table S4. The datasets supporting the conclusions of this article are available from the corresponding author upon reasonable request. The authenticity of this article was validated by uploading the key raw data onto the Research Data Deposit platform (www.researchdata.org.cn), and the number is 2301310001.
