# Supplementary material for: miR-30e-5p-mediated FOXD1 promotes cell proliferation by blocking cellular senescence and apoptosis through p21/CDK2/Rb signaling in head and neck carcinoma
Source: Cell Death Discov. 2023 Aug 10;9:295. doi: 10.1038/s41420-023-01571-2 (PMC10415393; doi:10.1038/s41420-023-01571-2)
Supplement: Supplementary file 1 — Supplementary Materials-Figures [file 41420_2023_1571_MOESM1_ESM.doc]

**Supplementary Materials-Figures**


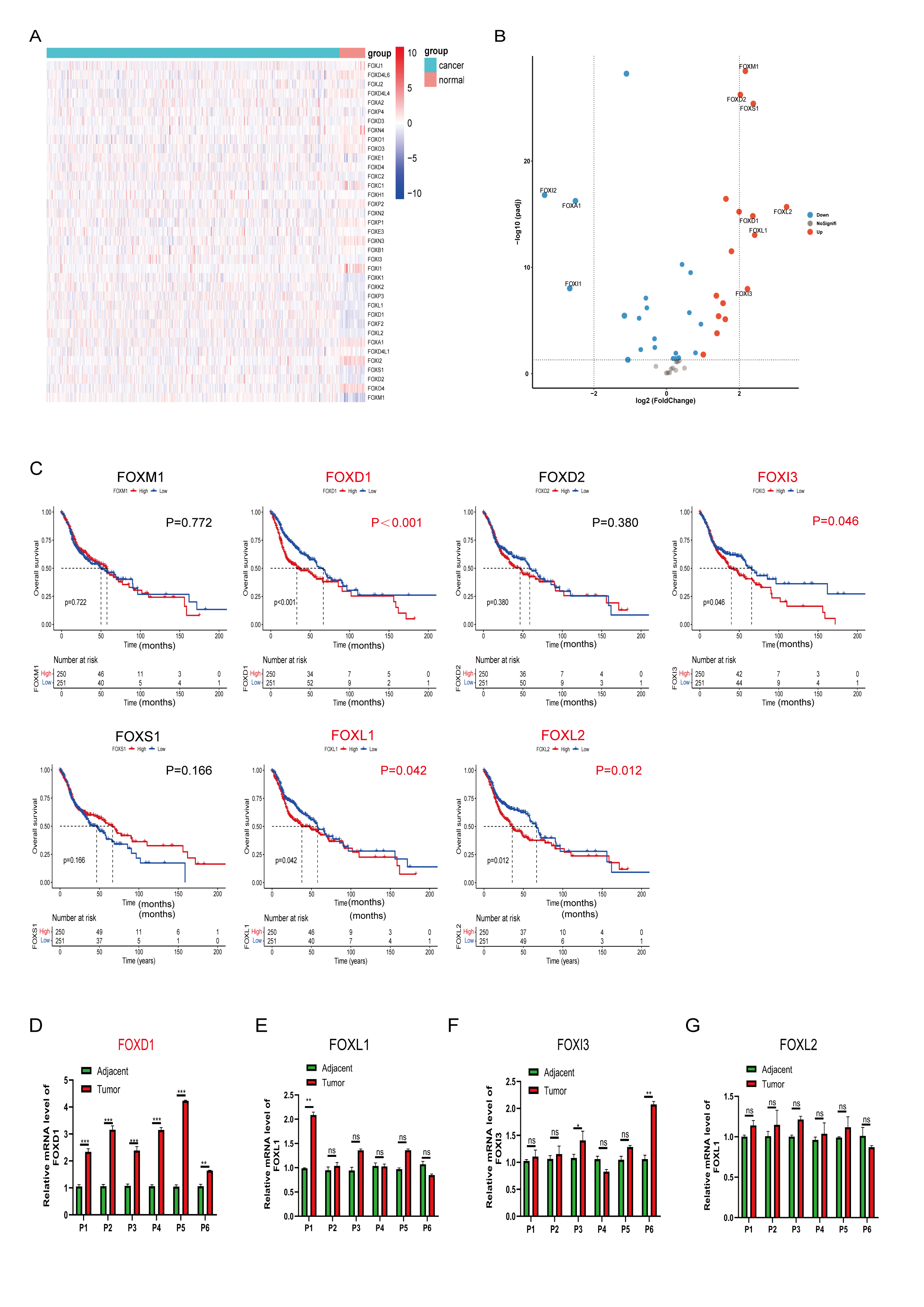


**Figure S1. FOXD1 is highly expressed in HNSCC.**

(**A**) Most FOX family genes were highly expressed in HNSCC tissues compared to normal tissues. (**B**) FOXD1, FOXD2, FOXM1, FOXS1, FOXL1, FOXL2, and FOXI3 were significantly overexpressed in HNSCC tissues compared to normal tissues (logFC>2). (**C**) Survival curve analysis revealed that FOXD1, FOXI3, FOXL1, and FOXL2 significantly affected the OS of patients (p < 0.05). (**D-G**) mRNA levels of FOXD1, FOXI3, FOXL1, and FOXL2 in HNSCC tissues compared to corresponding adjacent normal tissues. FOXD1, forkhead box D1; HNSCC, head and neck squamous cell carcinoma; OS, overall survival; mRNA, messenger RNA.


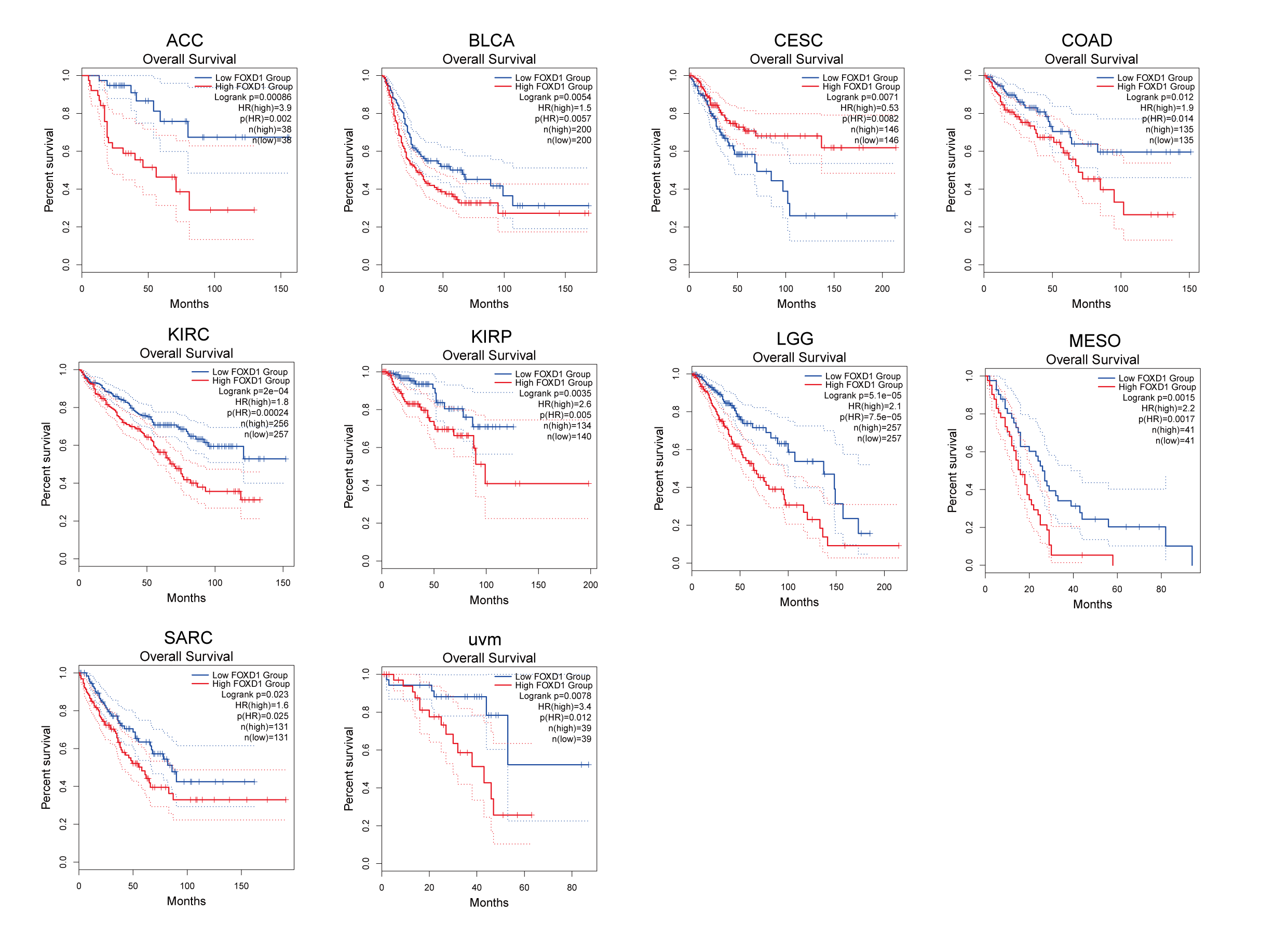


**Figure S2. FOXD1 survival curve analysis.** FOXD1 corresponds to poor OS in many cancers. FOXD1, forkhead box D1; OS, overall survival.

ACC，Adrenocortical carcinoma; BLCA, Bladder Urothelial Carcinoma; CESC, Cervical squamous cell carcinoma and endocervical adenocarcinoma; COAD, Colon adenocarcinoma；KIRC, Kidney renal clear cell carcinoma; KIRP, Kidney renal papillary cell carcinoma; LGG, Brain Lower Grade Glioma; MESO, Mesothelioma; SARC, Sarcoma; UVM, Uveal Melanoma.


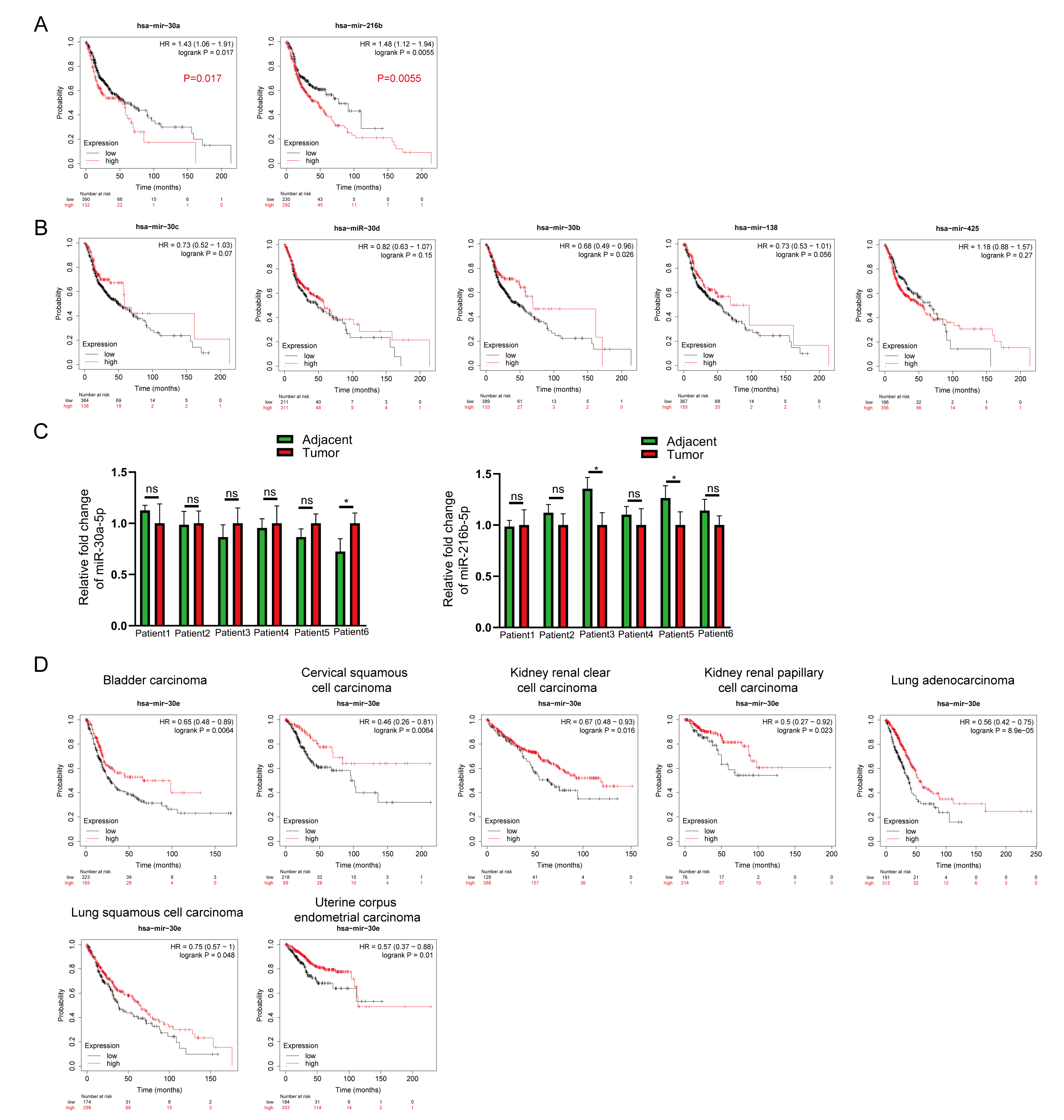


*Figure. S3* **miR-30e-5p predicts better prognosis in some types of cancers.***.*

(A) Survival curves of mir-30a and mir-216b in HNSCC. (B) Survival curves of mir-30e, mir-30d, mir-30b, mir-138, and mir-425 in HNSCC. (C) Expression levels of miR-30a-5p and miR-216b-5p in six pairs of HNSCC tumor tissues and adjacent normal tissues. **(D)** Kaplan-Meier plotter databases indicated that miR-30e-5p predicted better prognosis in some types of cancers. HNSCC, head and neck squamous cell carcinoma.
