# Supplementary material for: miR-30e-5p-mediated FOXD1 promotes cell proliferation by blocking cellular senescence and apoptosis through p21/CDK2/Rb signaling in head and neck carcinoma
Source: Cell Death Discov. 2023 Aug 10;9:295. doi: 10.1038/s41420-023-01571-2 (PMC10415393; doi:10.1038/s41420-023-01571-2)
Supplement: Supplementary file 2 — Supplementary Materials-Tables [file 41420_2023_1571_MOESM2_ESM.doc]

**Table S1.** The primer sequences for qPCR assay.

| Gene/MicroRNA name | Primer Sequence |
| --- | --- |
| FOXD1 | FOXD1-up 5’-GATCTGTGAGTTCATCAGCGGC-3’  FOXD1-dn 5’-TGACGAAGCAGTCGTTGAGCGA-3’ |
| GAPDH | GAPDH-up 5’-GCACCGTCAAGGCTGAGAAC-3’  GAPDH-dn 5’-ATGGTGGTGAAGACGCCAGT-3’ |
| FOXI3 | FOXI3-up  5’-GCTAGCTCGAGATGACATCATTTGTTCCACAGTC-3’  FOXI3-dn 5’-GCTCTAGATTACACCTCAGATCCCTCCC-3’ |
| FOXL1 | FOXL1-up 5’-TTATTTGGCGGACAGTGACA-3’  FOXL1-dn 5’-ACACGGCATCAATCTTTTCC-3’ |
| FOXL2 | FOXL2-up 5’-GAGTTTTTGTTGGGCCTTCA-3’;  FOXL2-dn 5’-GAGGGTGAA ACTTCCCCA AT-3’ |
| P21 | P21-up 5’-AGGGGACAGCAGAGGAAGAC-3’  P21-dn 5’-GGCGTTTGGAGTGGTAGAAA-3’ |
| miR-216b-5p | miR-216b-5p-up 5’-GGGGAAATCTCTGCAGGCAA-3′  miR-216b-5p-dn 5′-CAGTGCAGGGTCCGAGGT-3′ |
| miR-30a-5p | miR-30a-5p-up 5’-TGTAAACATCCTCGAC-3’  miR-30a-5p-dn 5’-GTGCAGGGTCCGAGGT-3’ |
| miR-30e-5p | miR-30e-5p-up 5’-CGGGCTGTAAACATCCTTGAC-3’  miR-30e-5p-dn  5’GTCGTATCCAGTGCAGGGTCCGAGGTATTCGCACTGGATACGACCTTCCA-3’ |

**Table S2. The information and application of antibodies.**

| Gene name | Company name | Catalog number | Application |
| --- | --- | --- | --- |
| FOXD1 | Invitrogen | PA5-27142 | WB/ChiP |
| FOXD1 | Abclone | A20240 | IHC |
| P21 | Cell Signaling Technology | 2947S | WB/IHC |
| CDK4 | Cell Signaling Technology | 12790S |  |
| CDK6 | Cell Signaling Technology | 13331S | WB |
| CDK2 | Cell Signaling Technology | [18048](https://www.cellsignal.cn/products/primary-antibodies/cdk2-e8j9t-xp-rabbit-mab/18048?site-search-type=Products&N=4294956287&Ntt=cdk2&fromPage=plp)S | WB |
| p-CDK4 | Invitrogen | PA5-64482 | WB |
| p-CDK6 | Invitrogen | 711588 | WB |
| p-CDK2 | Invitrogen | MA5-33128 | WB/IHC |
| cyclinE1 | Abcam | ab33911 | WB |
| Rb | Abcam | ab181616 | WB |
| p-Rb | Abcam | ab184796 | WB/IHC |
| Ki67 | Abcam | ab15580 | IHC |
| a-tublin | Cell Signaling Technology | 2148S | WB |
| GAPDH | Abcam | ab8245 | WB |

**Table S3.** Correlation between FOXD1 expression and clinicopathological characteristics of HNSCC patients

|  |  | **FOXD1 IHC staining** | |  |
| --- | --- | --- | --- | --- |
| **Variable** | **HNSCC cohort**  **(N=136)** | **Low expression**  **(N=68)** | **High expression**  **(N=68)** | **p-value** |
| **Gender** |  |  |  | 0.286 |
| Male | 86 | 46 | 40 |  |
| Female | 50 | 22 | 28 |  |
| **Age** |  |  |  | 0.864 |
| <55 | 67 | 33 | 34 |  |
| ≥55 | 69 | 35 | 34 |  |
| **AJCC stage** |  |  |  | **0.01** |
| StageI-II | 66 | 36 | 30 |  |
| StageIII-IV | 70 | 32 | 38 |  |
| **T classification** |  |  |  | **0.001** |
| T1-T2 | 77 | 48 | 29 |  |
| T3-T4 | 59 | 20 | 39 |  |
| **N classification** |  |  |  | 0.059 |
| N0 | 73 | 42 | 31 |  |
| N+ | 63 | 26 | 37 |  |
| **Smoking** |  |  |  | 0.607 |
| Yes | 69 | 33 | 36 |  |
| NO | 67 | 35 | 32 |  |
| **Drinking** |  |  |  | 0.385 |
| YES | 79 | 37 | 42 |  |
| NO | 57 | 31 | 26 |  |
| **Relapse** |  |  |  | **0.022** |
| Yes | 53 | 20 | 33 |  |
| No | 83 | 48 | 35 |  |

AJCC, American Joint Committee on Cancer; IHC, Immunohistochemistry.

**Table S4.** Univariate and multivariate analysis of factors associated with

OS survival in HNSCC patients.

|  | **Univariate analysis** | | | **Multivariate analysis** | | |
| --- | --- | --- | --- | --- | --- | --- |
| **Variate** | HR (95% CI) | P value | | HR (95% CI) | P value | |
| **Gender** | | | |  | | |
| Male | 1(Ref) |  | |  | | |
| Female | 0.663(0.403, 1.029) | 0.107 | |  | | |
| **Age** |  |  | |  | | |
| ≤55 | 1(Ref) |  | |  | | |
| >55 | 1.288(0.809, 2.049) | 0.286 | |  | | |
| **Drinking** |  | |  |  | | |
| No | 1(Ref) | |  |  | | |
| Yes | 1.119(0.746, 1.903) | | 0.464 |  | | |
| **Smoking** |  | |  |  | | |
| No | 1(Ref) | |  |  | | |
| Yes | 1.247(0.785, 1.981) | | 0.349 |  | |  |
| **N classification** |  | |  |  | | |
| N0 | 1(Ref.) | |  | 1(Ref.) | | |
| N+ | 1.780(1.117, 2.834) | | 0.015 | 1.363(0.820, 2.263) | 0.232 | |
| **T classification** |  | |  |  |  |  |
| T1-T2 | 1(Ref.) | |  | 1(Ref.) | | |
| T3-T4 | 1.901(1.119, 3.026) | | 0.007 | 1.410(0.869, 2.287) | 0.164 | |
| **AJCC Stage** |  | |  |  | | |
| Stage I-II | 1(Ref.) | |  | 1(Ref.) | | |
| Stage III-IV | 1.881(1.170, 3.022) | | 0.009 | 1.584(0.944, 2.658) | 0.082 | |
| **FOXD1 expression** |  | |  |  | | |
| Low | 1(Ref.) | |  | 1(Ref.) | | |
| High | 2.215(1.385, 3.542) | | 0.001 | 2.039(1.255，3.313) | **0.004** | |

AJCC, American Joint Committee on Cancer; OS, ovrall survival; HR, hazard ratio. Ref., reference.

**Table S5.** Univariate and multivariate analysis of factors associated with

DFS survival in HNSCC patients.

|  | **Univariate analysis** | | | **Multivariate analysis** | | |
| --- | --- | --- | --- | --- | --- | --- |
| **Variate** | HR (95% CI) | P value | | HR (95% CI) | P value | |
| **Gender** | | | |  | | |
| Male | 1(Ref) |  | |  | | |
| Female | 0.661(0.402, 1.086) | 0.102 | |  | | |
| **Age** |  |  | |  | | |
| ≤55 | 1(Ref) |  | |  | | |
| >55 | 1.345(0.844, 2.141) | 0.212 | |  | | |
| **Drinking** |  | |  |  | | |
| No | 1(Ref) | |  |  | | |
| Yes | 1.114(0.698, 1.779) | | 0.650 |  | | |
| **Smoking** |  | |  |  | | |
| No | 1(Ref) | |  |  | | |
| Yes | 1.197 (0.754, 1.901) | | 0.447 |  | |  |
| **N classification** |  | |  |  | | |
| N0 | 1(Ref.) | |  | 1(Ref.) | | |
| N+ | 1.827(1.114, 2.912) | | 0.011 | 1.327(0.783, 1.327) | 0.294 | |
| **T classification** |  | |  |  |  |  |
| T1-T2 | 1(Ref.) | |  | 1(Ref.) | | |
| T3-T4 | 1.937(1.216, 3.086) | | 0.005 | 1.412(0.863, 2.308) | 0.169 | |
| **AJCC Stage** |  | |  |  | | |
| Stage I-II | 1(Ref.) | |  | 1(Ref.) | | |
| Stage III-IV | 1.870 (1.163, 3.005) | | 0.01 | 1.521(0.892, 2.594) | 0.123 | |
| **FOXD1 expression** |  | |  |  | | |
| Low | 1(Ref.) | |  | 1(Ref.) | | |
| High | 2.204(1.378, 3.523) | | 0.001 | 1.963(1.207，3.193) | **0.007** | |

AJCC, American Joint Committee on Cancer; DFS, disease-free survival; HR, hazard ratio. Ref., reference.

**Table S6.** Predicted consequential pairing of target region (www.targetscan.org).

| Position 513-520 of FOXD1 3' UTR | Predicted consequential pairing of target region (top) and miRNA (bottom) |
| --- | --- |
| hsa-miR-30d-5p | 5' ...GAAAAAUGGUUAACAUGUUUACA...  |||||||  3' GAAGGUCAGCCCCUACAAAUGU |
| hsa-miR-30b-5p | 5' ...GAAAAAUGGUUAACAUGUUUACA...  |||||||  3' UCGACUCACAUCCUACAAAUGU |
| hsa-miR-30c-5p | 5' ...GAAAAAUGGUUAACAUGUUUACA...  |||||||  3' CGACUCUCACAUCCUACAAAUGU |
| hsa-miR-30e-5p | 5' ...GAAAAAUGGUUAACAUGUUUACA...  |||||||  3' GAAGGUCAGUUCCUACAAAUGU |
| hsa-miR-30a-5p | 5' ...GAAAAAUGGUUAACAUGUUUACA...  |||||||  3' GAAGGUCAGCUCCUACAAAUGU |
| hsa-miR-216b-5p | 5' ...GCCUUUUUGAGGUGUAGAGAUUC...  |||||||  3' AGUGUAAACGGACGUCUCUAAA |
| hsa-miR-138-5p | 5' ...UGAACAAACAUUGGCCACCAGCU...  |||||||  3' GCCGGACUAAGUGUUGUGGUCGA |
| hsa-miR-425-5p | 5' ...CUUGUACCACACAUGGUGUCAUG...  |||||||  3' AGUUGCCCUCACUAGCACAGUAA |
