# Supplementary material for: miR-30e-5p-mediated FOXD1 promotes cell proliferation by blocking cellular senescence and apoptosis through p21/CDK2/Rb signaling in head and neck carcinoma
Source: Cell Death Discov. 2023 Aug 10;9:295. doi: 10.1038/s41420-023-01571-2 (PMC10415393; doi:10.1038/s41420-023-01571-2)
Supplement: Supplementary file 3 — Original Data File [file 41420_2023_1571_MOESM3_ESM.doc]

***Original strips in western-blot assay of the research***

***Figure 1***

Figure1C GAPDH
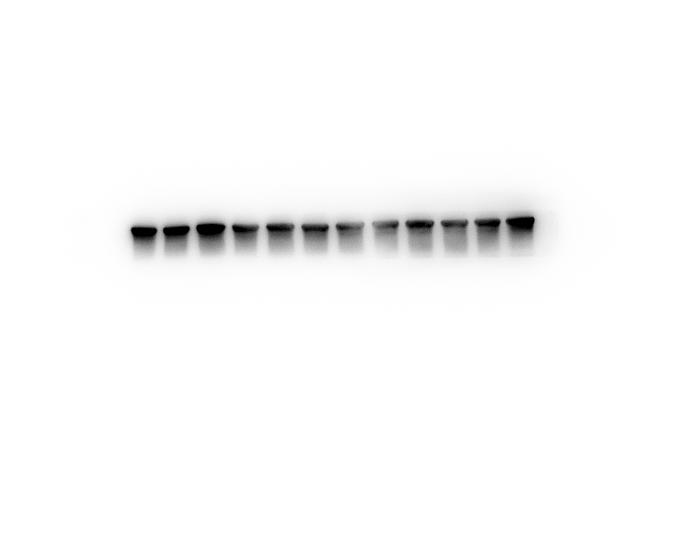


-35kda

Figure1C FOXD1
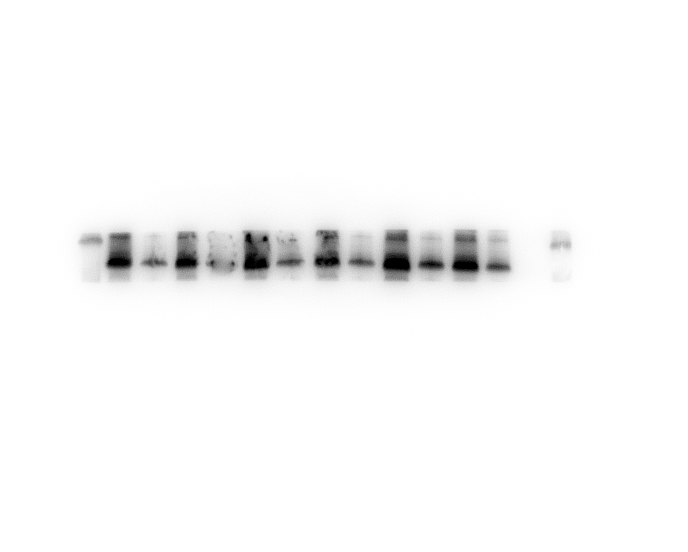


-45kda

Figure1D GAPDH
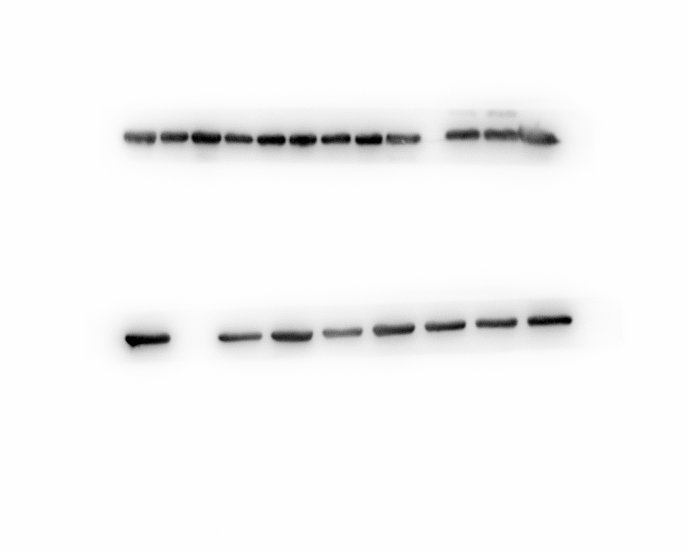


-35kda

Figure1D FOXD1
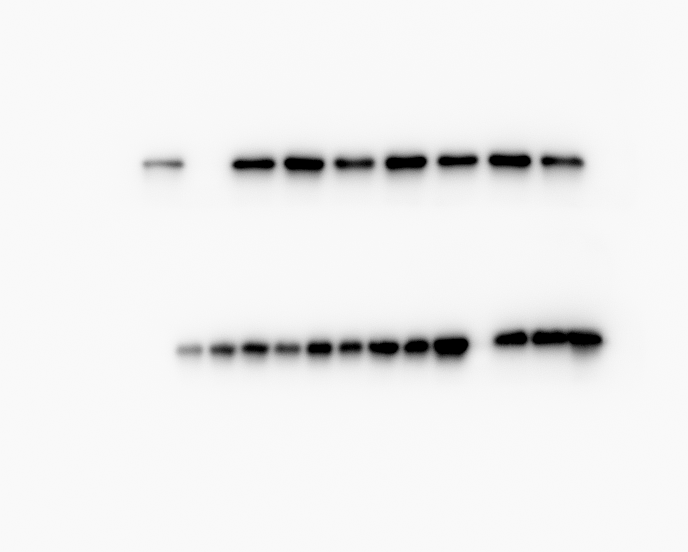


-45kda

***Figure 2***

Figure2A GAPDH (SCC25 sh-FOXD1-NC, sh-FOXD1#1, sh-FOXD1#3)
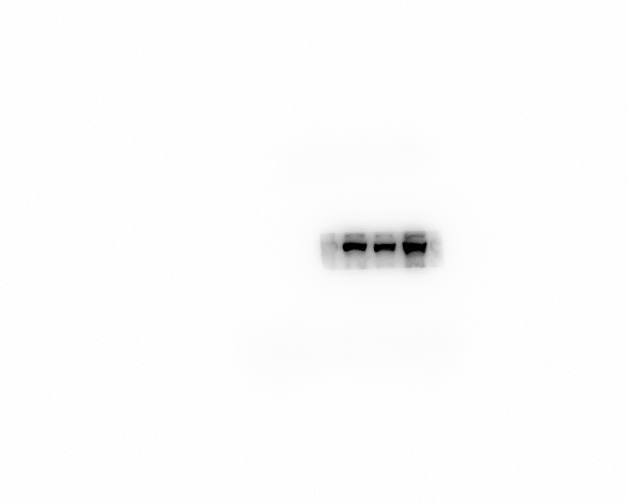


-35kda

Figure2A FOXD1(SCC25 sh-FOXD1-NC, sh-FOXD1#1, sh-FOXD1#3)
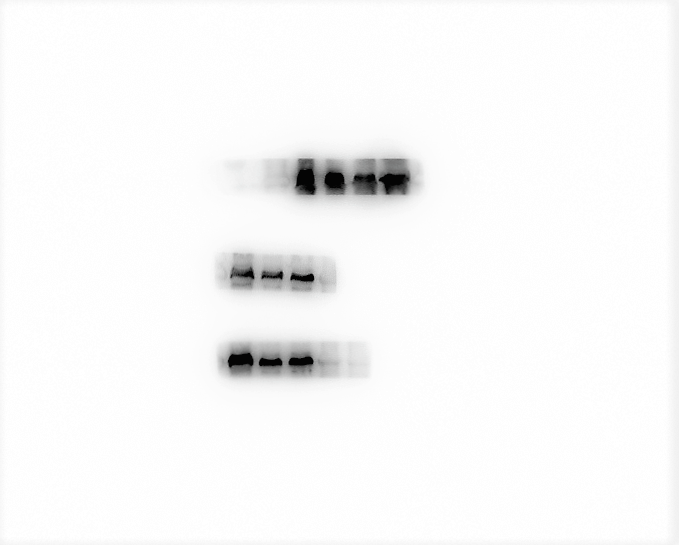


-45kda

Figure2A GAPDH(SCC25 Vector, OE)
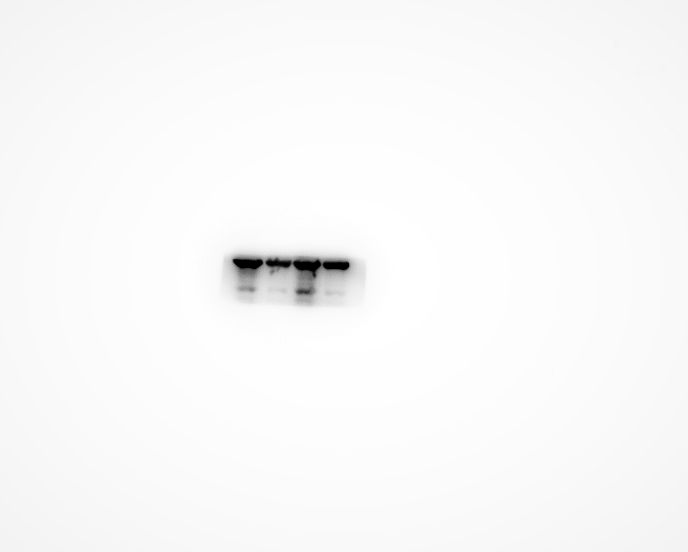


-35kda

Figure2A FOXD1(SCC25 Vector, OE)
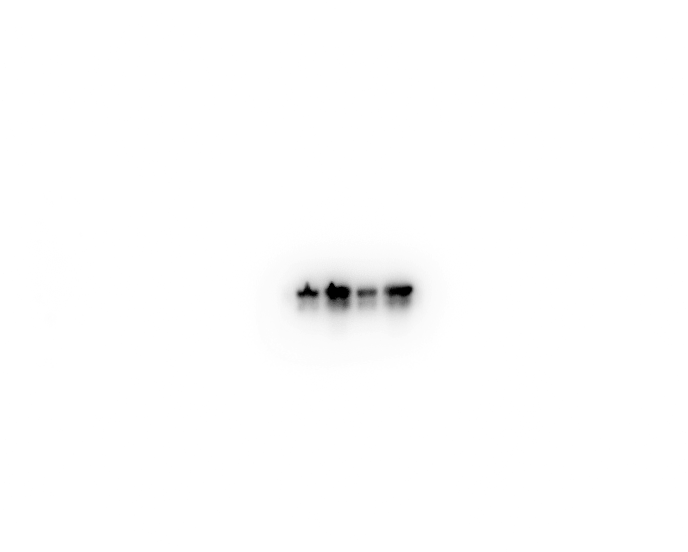


-45kda

Figure2A GAPDH (Fadu sh-FOXD1-NC, sh-FOXD1#1, sh-FOXD1#3)
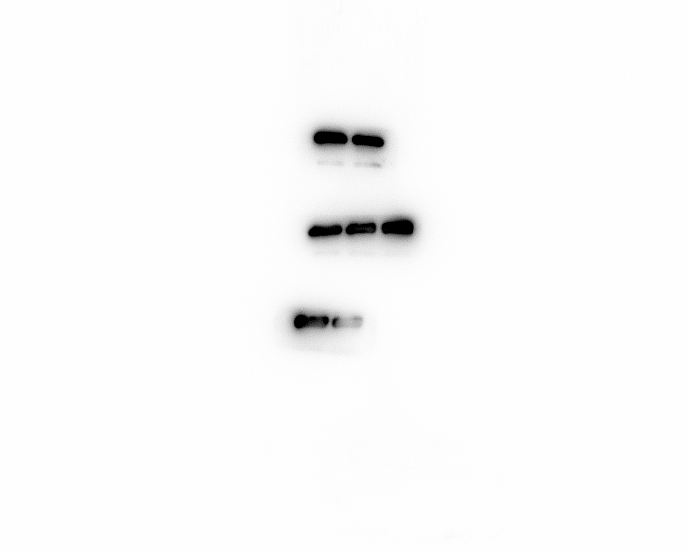


-35kda

Figure2A FOXD1 (Fadu sh-FOXD1-NC, sh-FOXD1#1, sh-FOXD1#3)
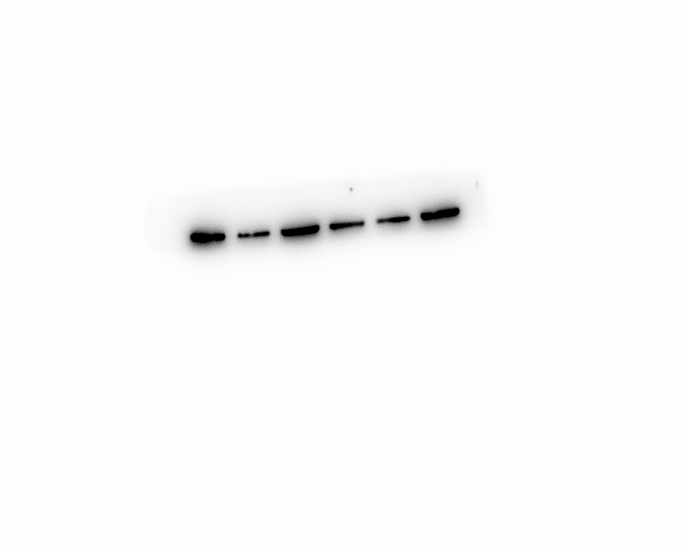


-45kda

Figure2A GAPDH(Fadu Vector, OE)
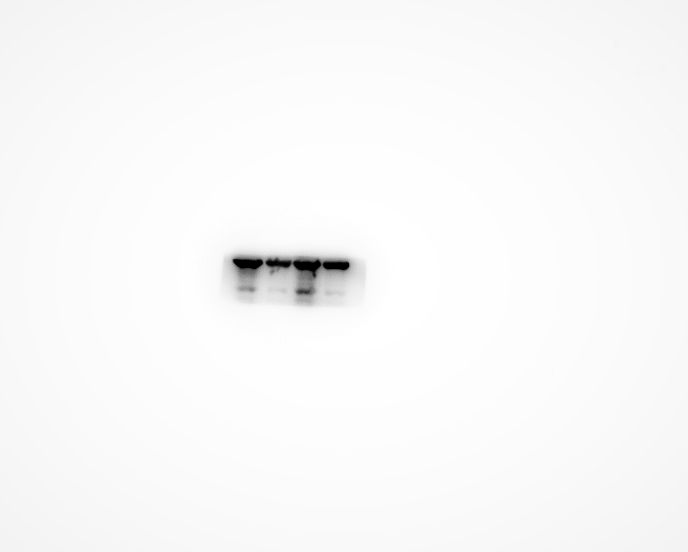


-35kda

Figure2A FOXD1(Fadu Vector, OE)
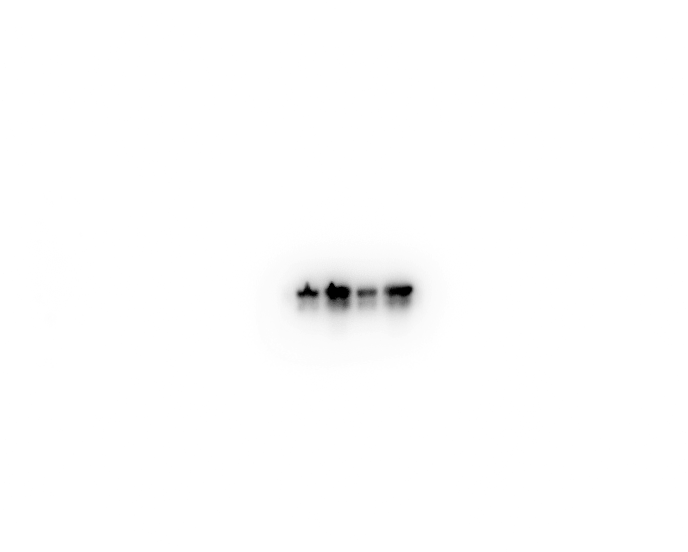


-45kda

***Figure 3***

Figure3E FOXD1(SCC25 sh-FOXD1-NC, sh-FOXD1#1, sh-FOXD1#3)
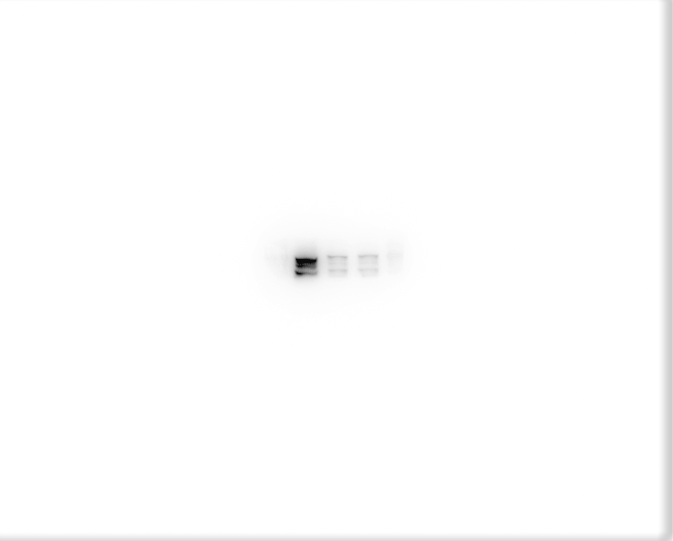


-45kda

Figure3E FOXD1(SCC25 Vector, OE)
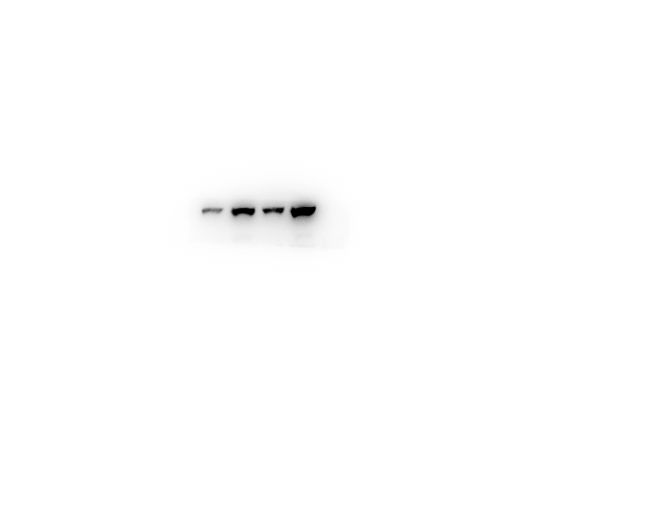


-45kda

Figure3E p21(SCC25 sh-FOXD1-NC, sh-FOXD1#1, sh-FOXD1#3)
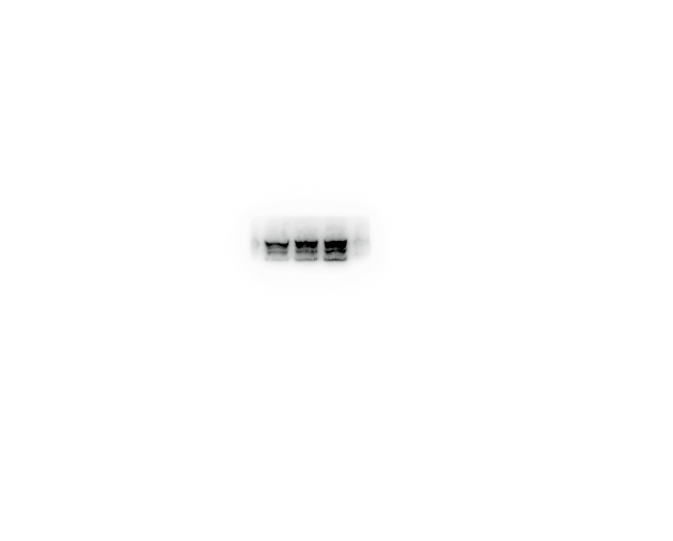


-20kda

Figure3E p21(SCC25 Vector, OE)
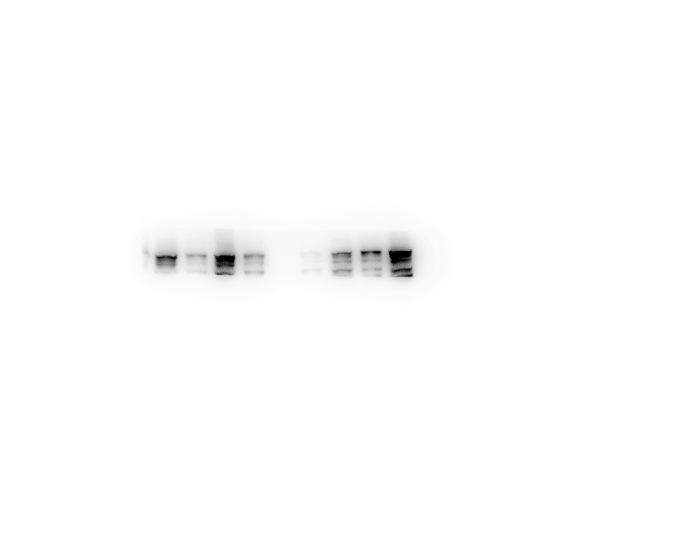


-20kda

Figure3E GAPDH(SCC25 sh-FOXD1-NC, sh-FOXD1#1, sh-FOXD1#3)
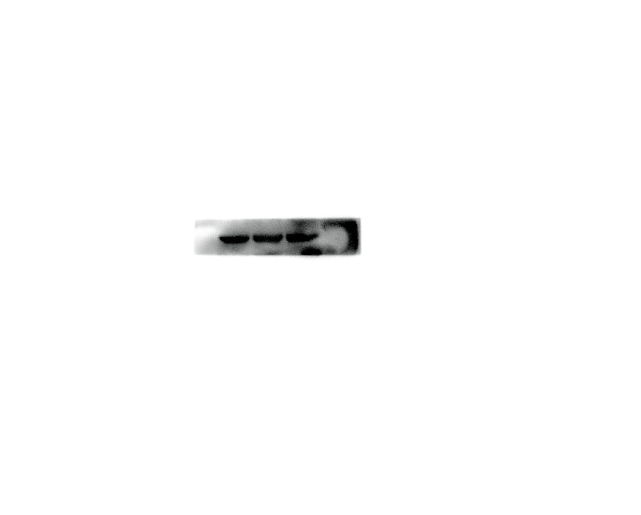


-35kda

Figure3E GAPDH(SCC25 Vector, OE)
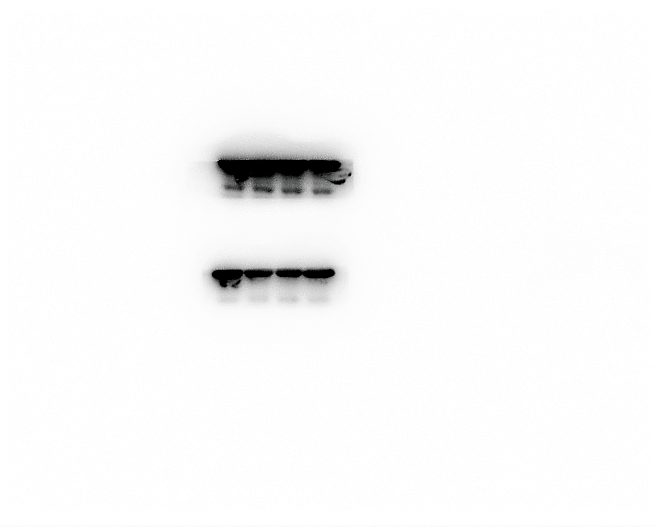


-35kda

Figure3E FOXD1(Fadu sh-FOXD1-NC, sh-FOXD1#1, sh-FOXD1#3)
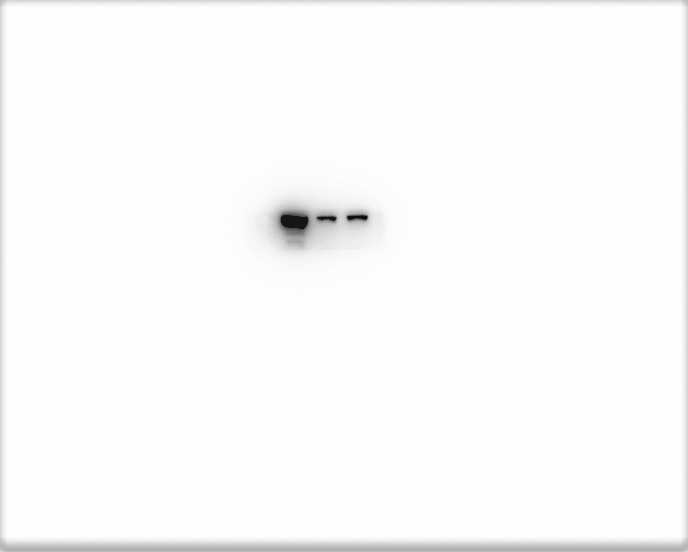


-45kda

Figure3E FOXD1(Fadu Vector, OE)
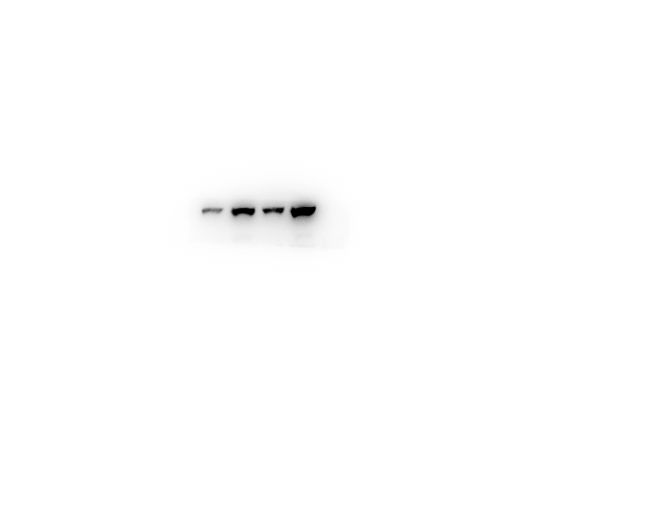


-45kda

Figure3E p21(Fadu sh-FOXD1-NC, sh-FOXD1#1, sh-FOXD1#3)
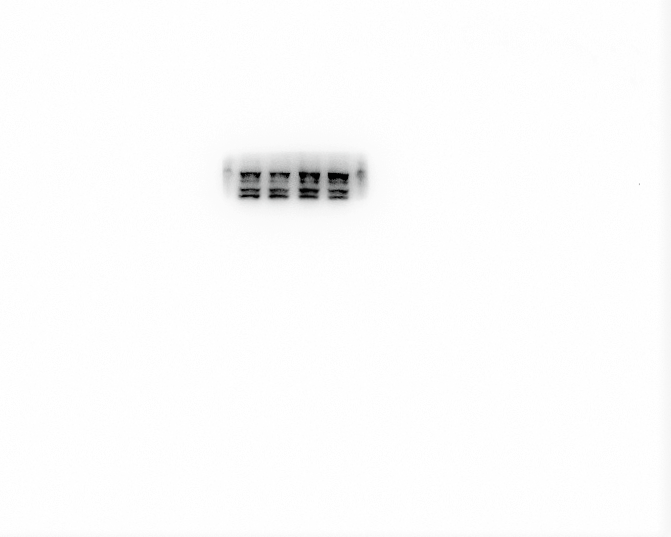


-20kda

Figure3E p21(Fadu Vector, OE)
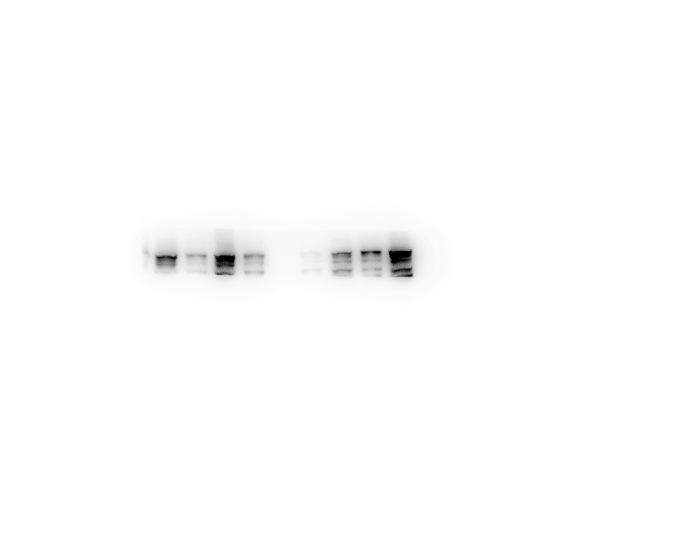


-20kda

Figure3E GAPDH(Fadu sh-FOXD1-NC, sh-FOXD1#1, sh-FOXD1#3)
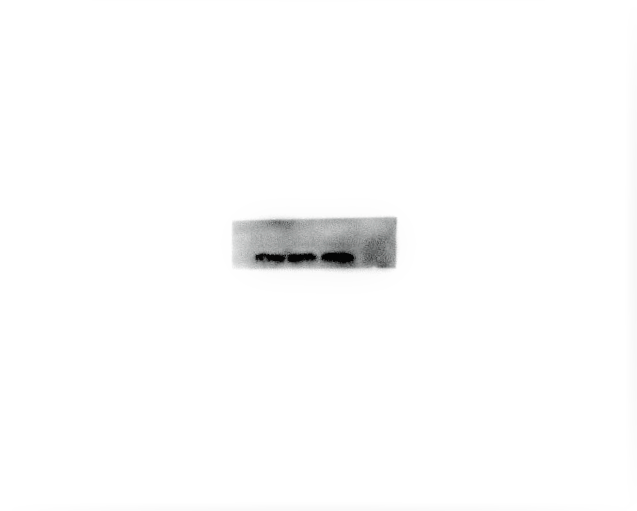


-35kda

Figure3E GAPDH (Fadu Vector, OE)
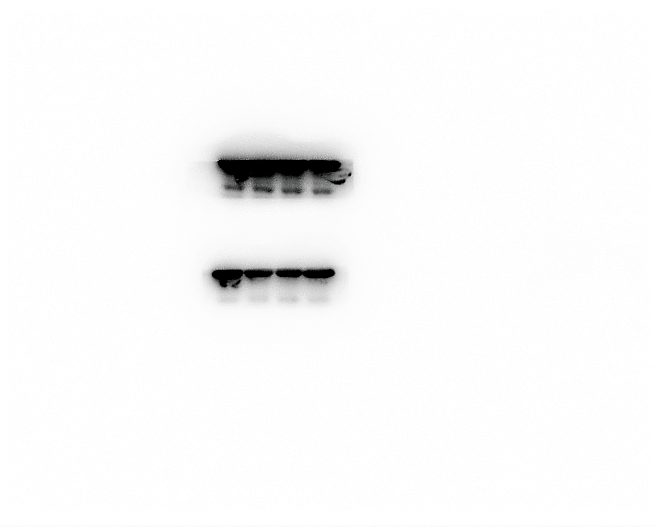


-35kda

***Figure 4***

Figure4A FOXD1 (SCC25)
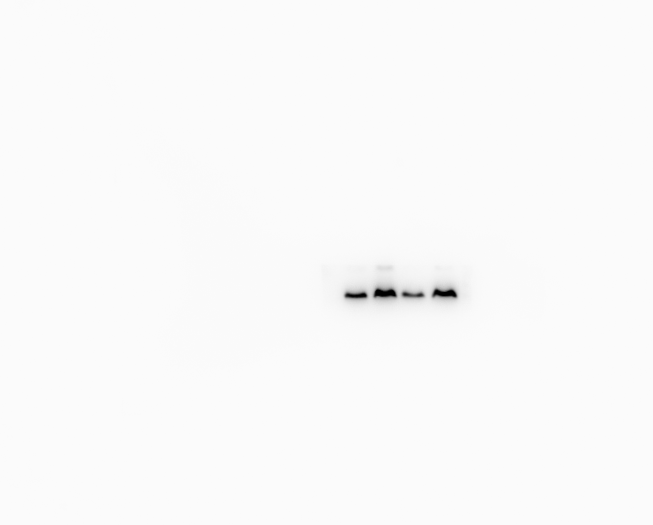


-45kda

Figure4A FOXD1 (Fadu)
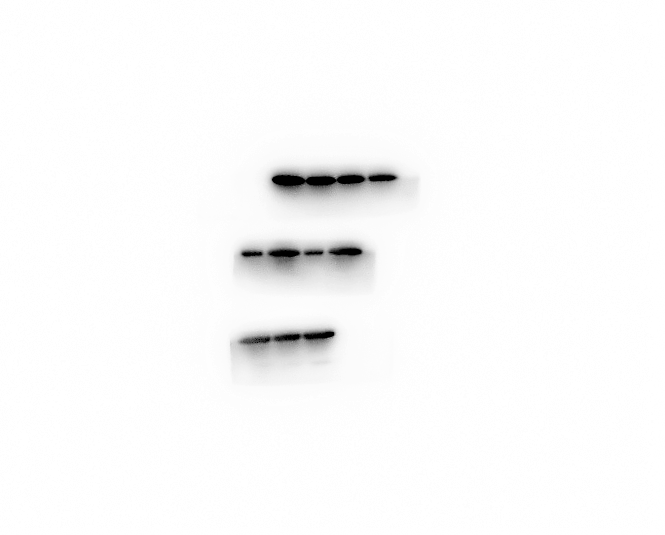


-45kda

Figure4A p21 (SCC25)
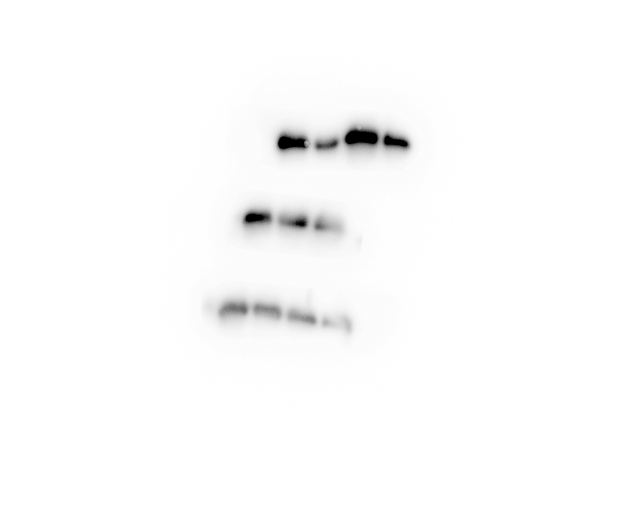


-20kda

Figure4A p21 (Fadu)
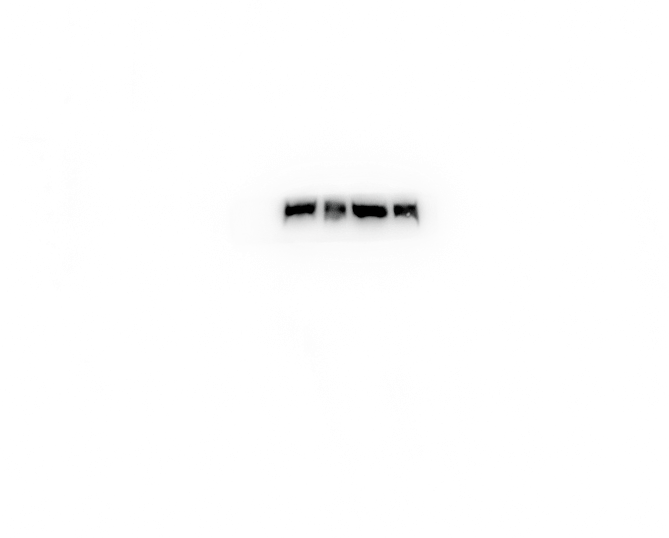


-20kda

Figure4A GAPDH (SCC25)
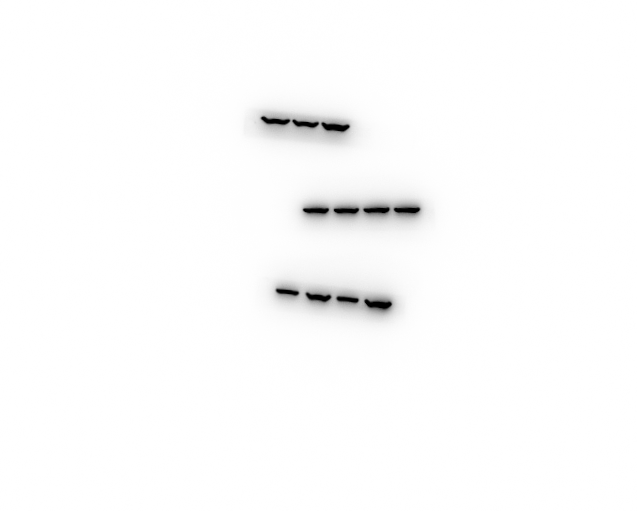


-35kda

-35kda

Figure4A GAPDH (Fadu)
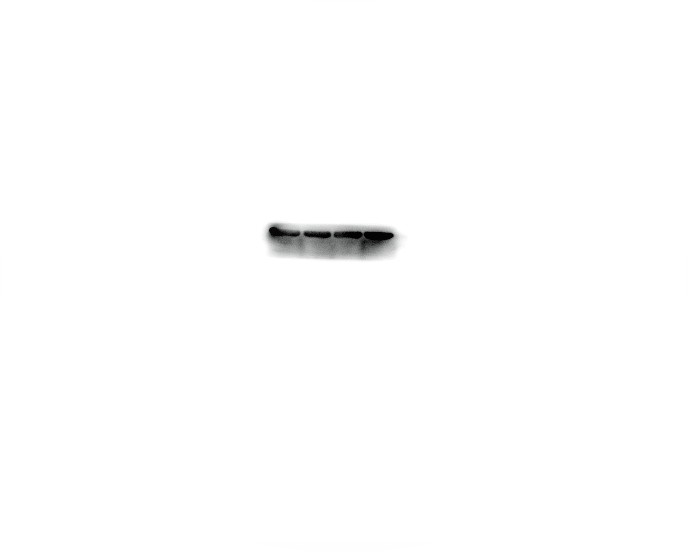


***Figure 6***

Figure6A

FOXD1 (SCC25 sh-FOXD1-NC, sh-FOXD1#1, sh-FOXD1#3); FOXD1 (Vector, OE)


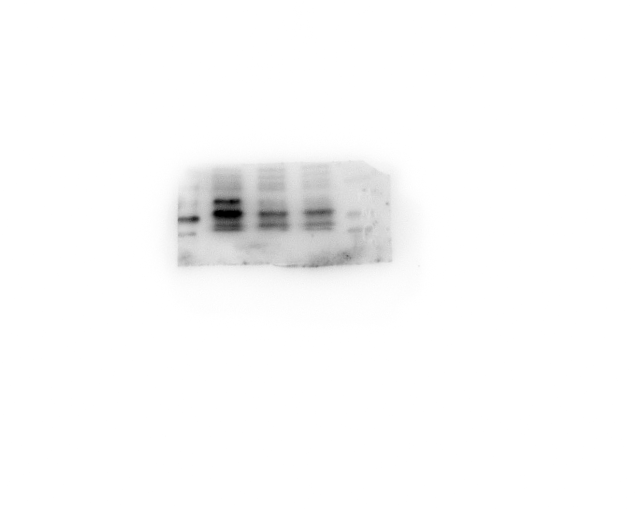

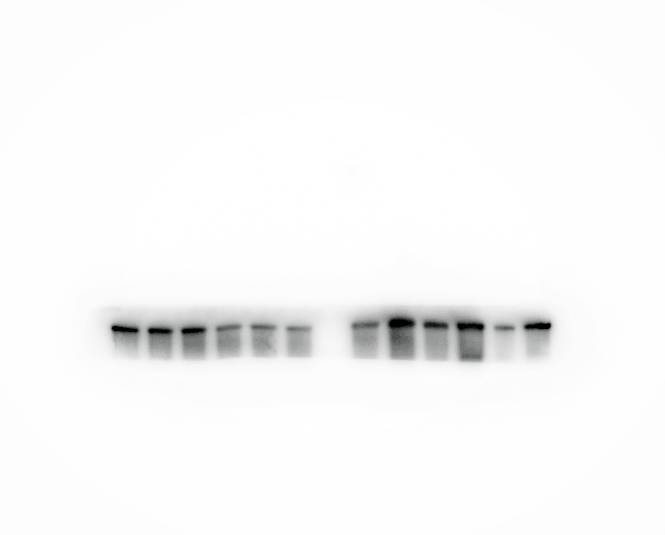


-45kda

-45kda

p21(SCC25 sh-FOXD1-NC, sh-FOXD1#1, sh-FOXD1#3); p21 (Vector, OE)


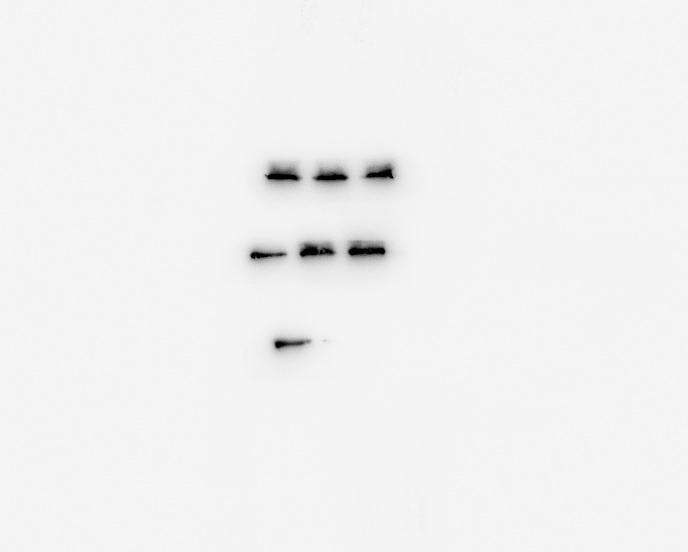

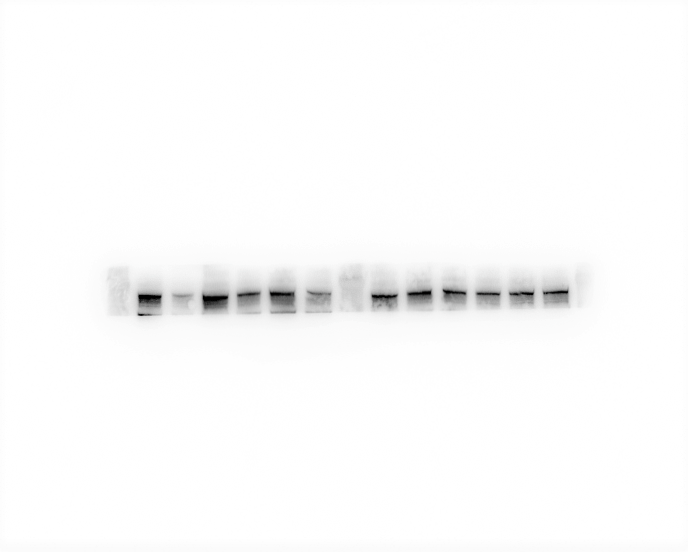


-20kda

-20kda

CDK4(SCC25 sh-FOXD1-NC, sh-FOXD1#1, sh-FOXD1#3); CDK4(Vector, OE)


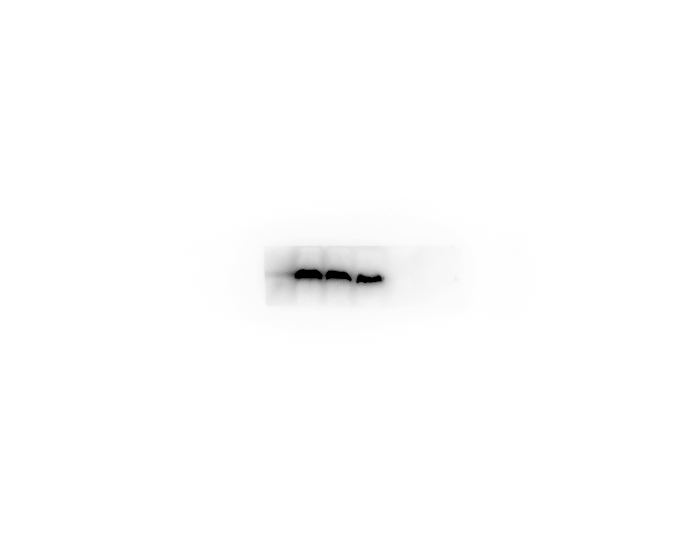

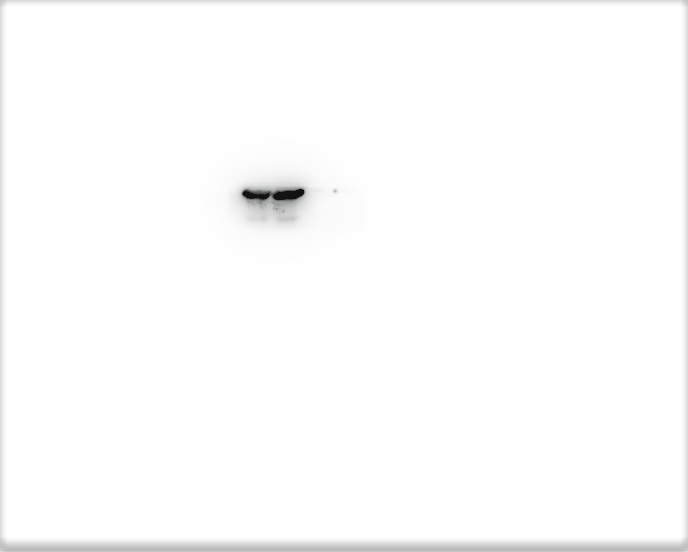


-35kda

-35kda

p-CDK4(SCC25 sh-FOXD1-NC, sh-FOXD1#1, sh-FOXD1#3); p-CDK4(Vector, OE)


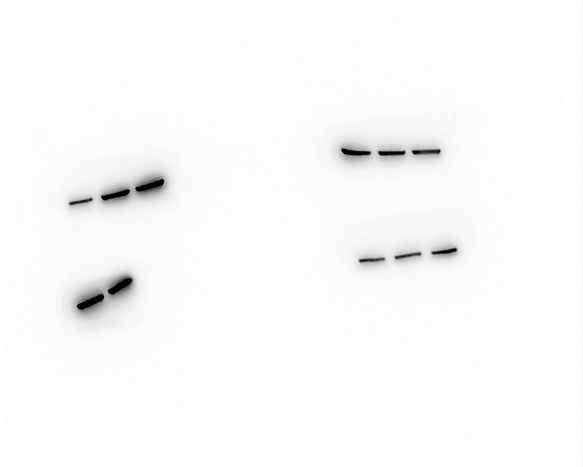

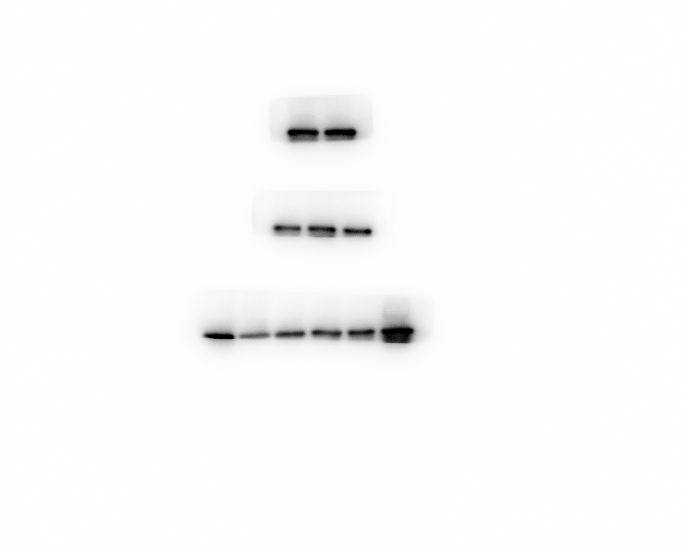


-35kda

-35kda

CDK6(SCC25 sh-FOXD1-NC, sh-FOXD1#1, sh-FOXD1#3); CDK6(Vector, OE)


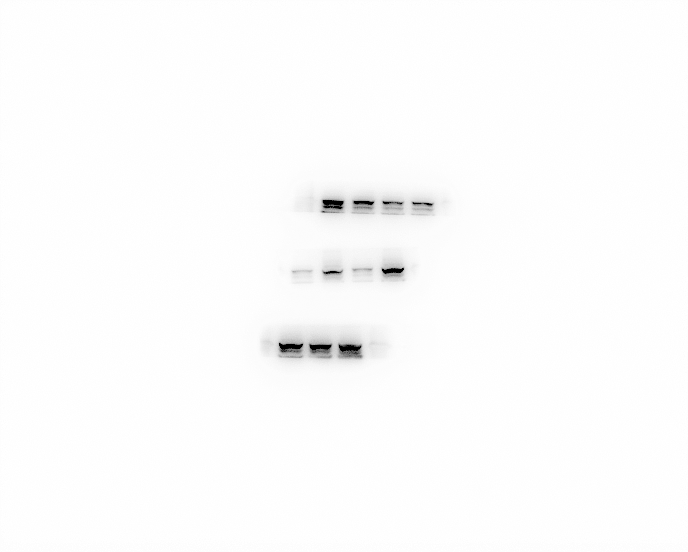

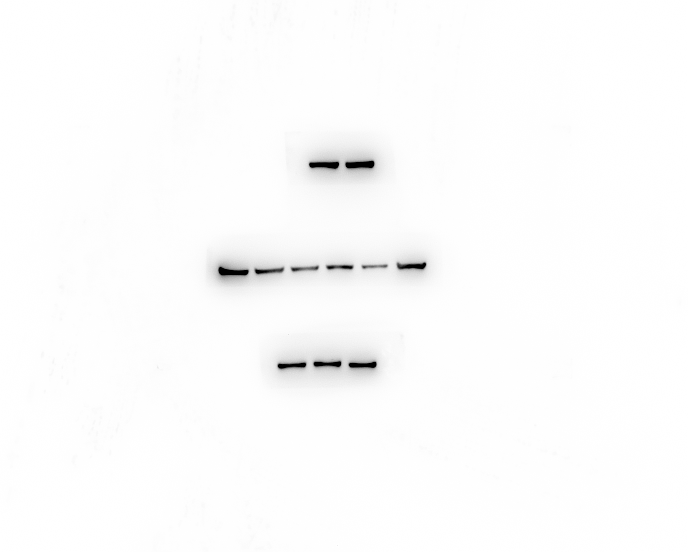


-35kda

-35kda

p-CDK6(SCC25 sh-FOXD1-NC, sh-FOXD1#1, sh-FOXD1#3); p-CDK6(Vector, OE)


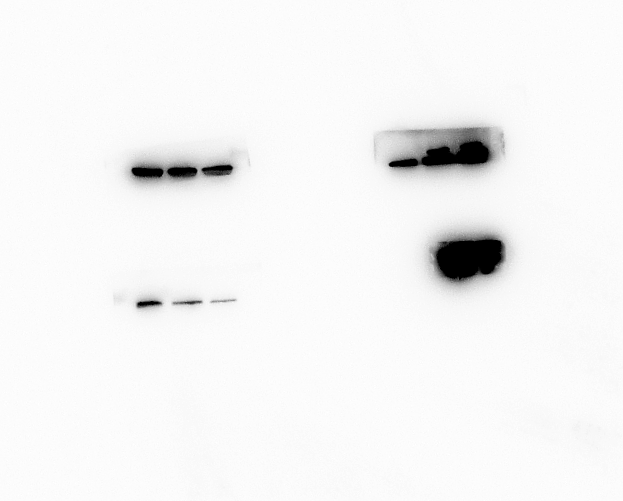

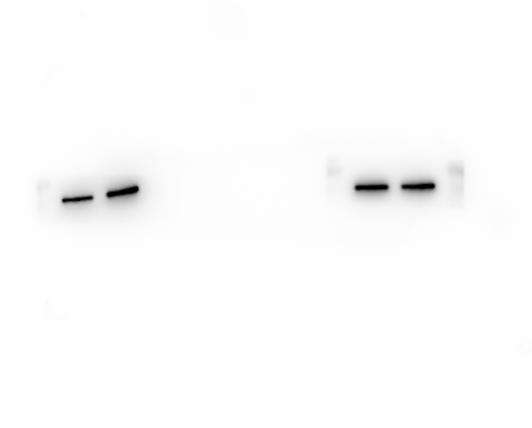


-35kda

-35kda

CDK2(SCC25 sh-FOXD1-NC, sh-FOXD1#1, sh-FOXD1#3); CDK2(Vector, OE)


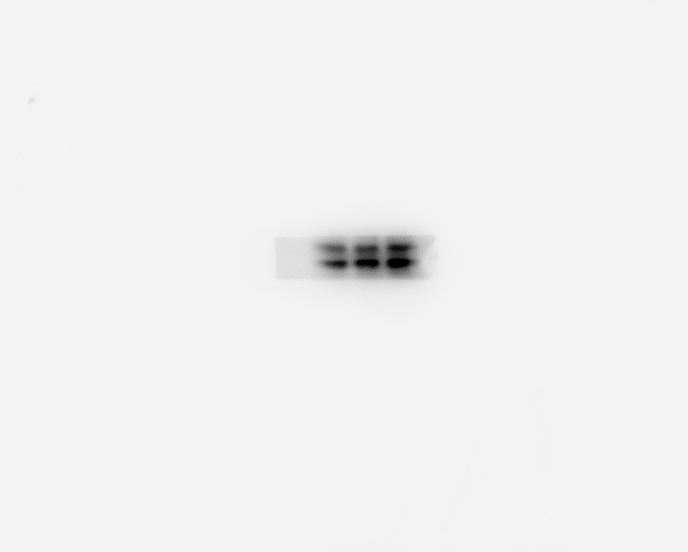

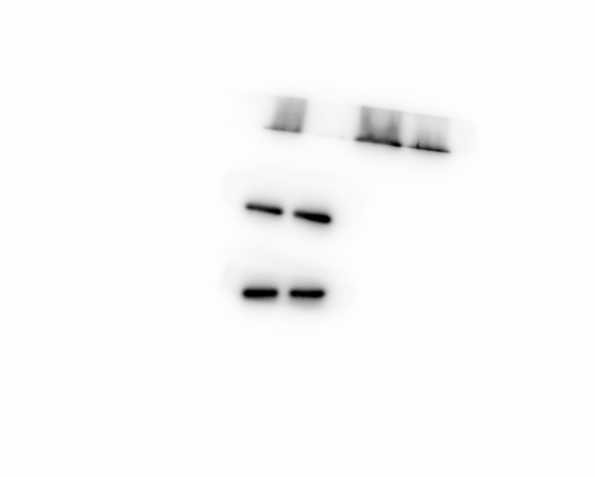


-35kda

-35kda

p-CDK2(SCC25 sh-FOXD1-NC, sh-FOXD1#1, sh-FOXD1#3); p-CDK2(Vector, OE)


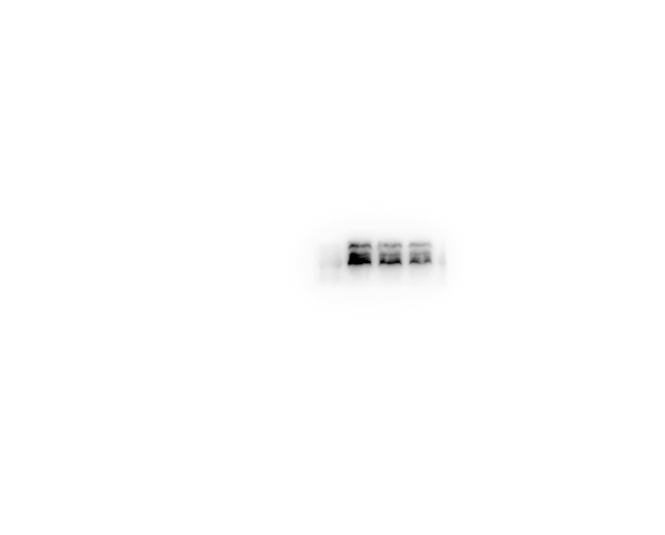

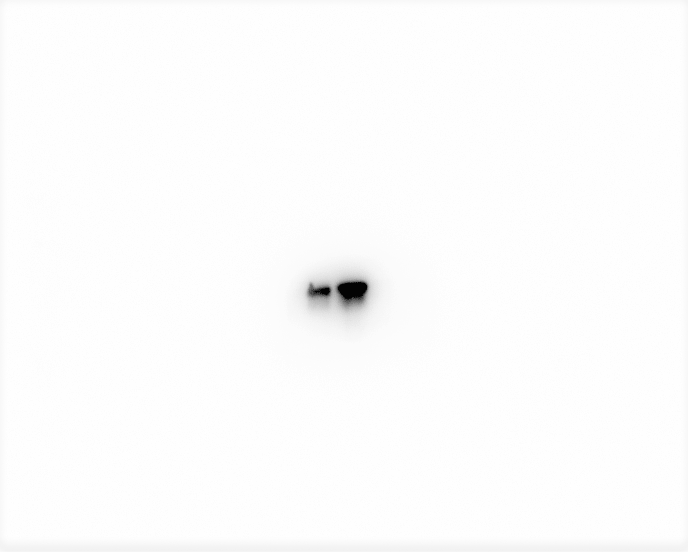


-35kda

-35kda

cyclinE1(SCC25 sh-FOXD1-NC, sh-FOXD1#1, sh-FOXD1#3); cyclinE1 (Vector, OE)


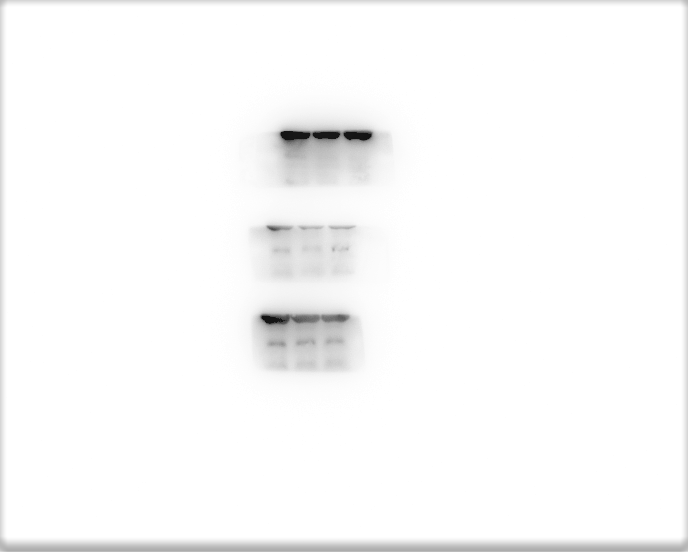

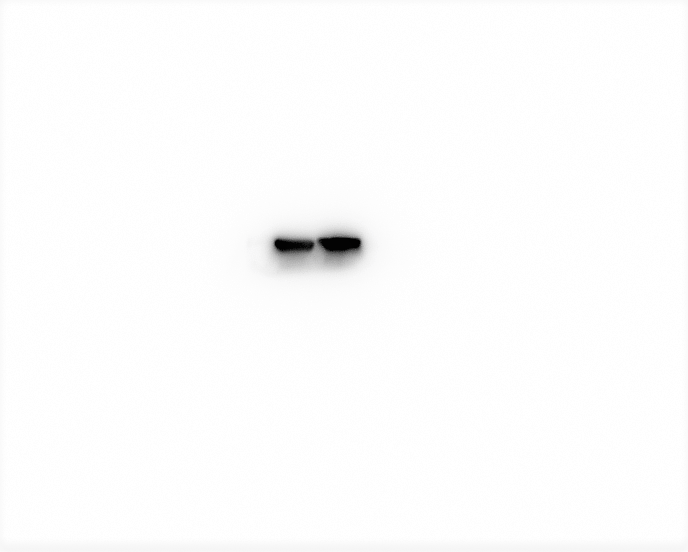


-45kda

-45kda

Rb(SCC25 sh-FOXD1-NC, sh-FOXD1#1, sh-FOXD1#3); Rb (Vector, OE)


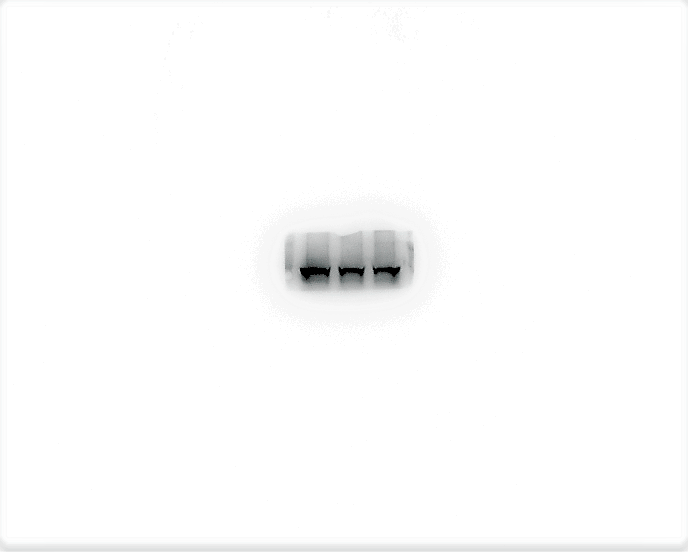

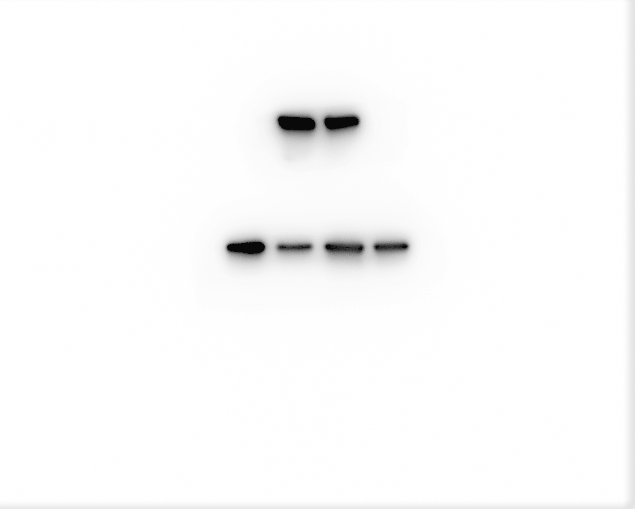


-100kdaa

-100kdaa

p-Rb(SCC25 sh-FOXD1-NC, sh-FOXD1#1, sh-FOXD1#3); p-Rb (Vector, OE)


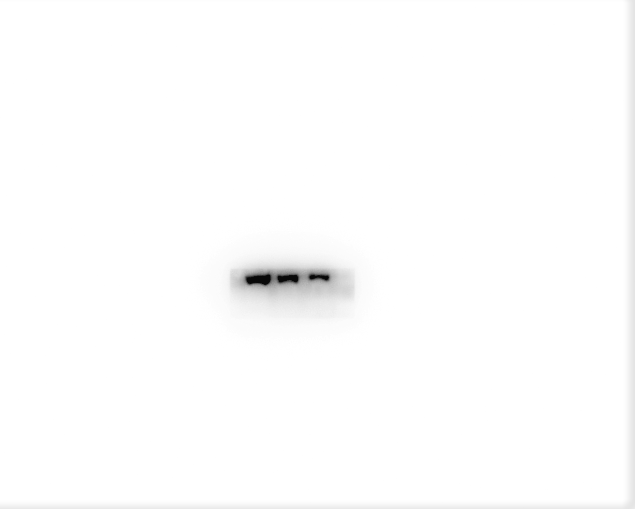

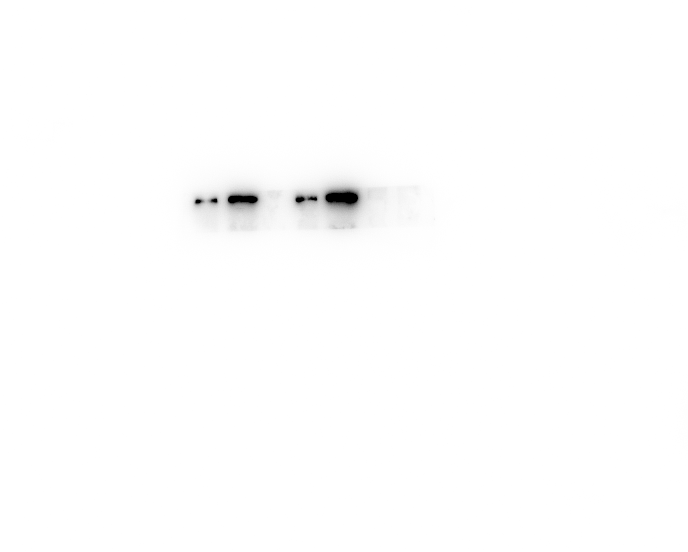


-100kdaa

-100kdaa

α-tublin(SCC25 sh-FOXD1-NC, sh-FOXD1#1, sh-FOXD1#3); α-tublin (Vector, OE)


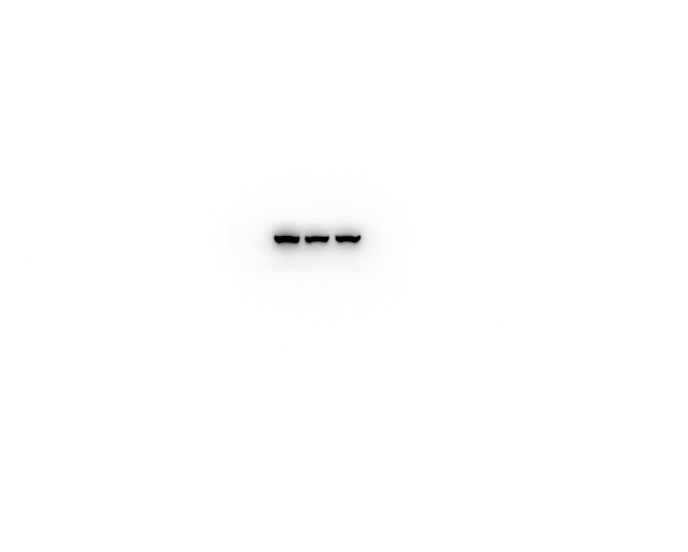

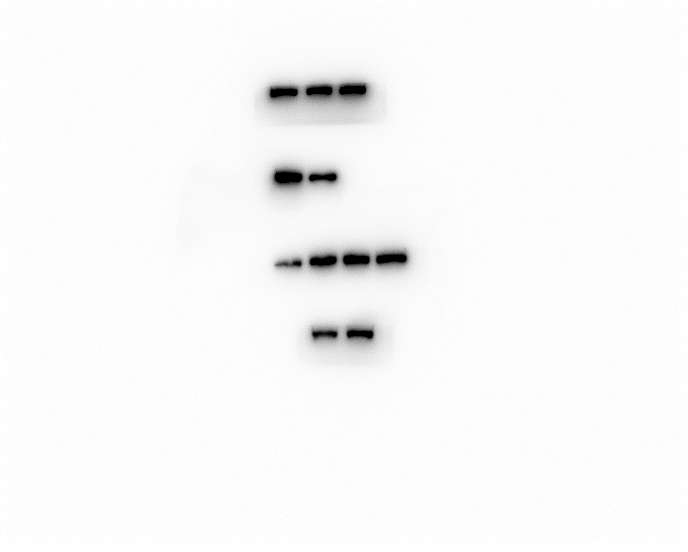


-45kda

-45kda

FOXD1 (Fadu sh-FOXD1-NC, sh-FOXD1#1, sh-FOXD1#3); FOXD1 (Vector, OE)


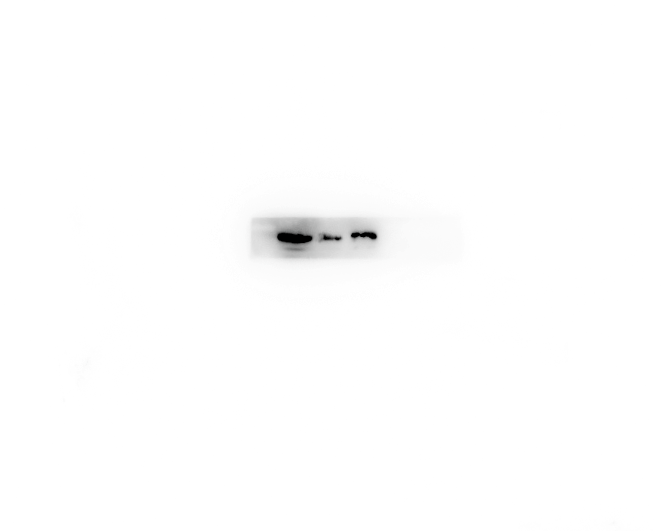

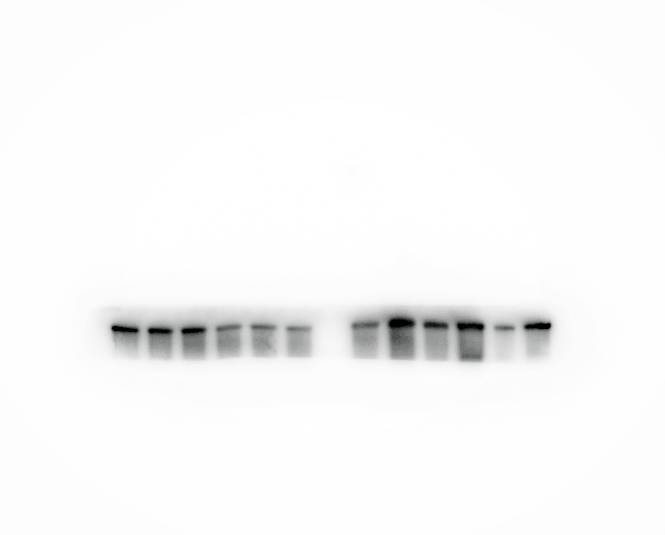


-45kda

-45kda

P21 (Fadu sh-FOXD1-NC, sh-FOXD1#1, sh-FOXD1#3); P21 (Vector, OE)


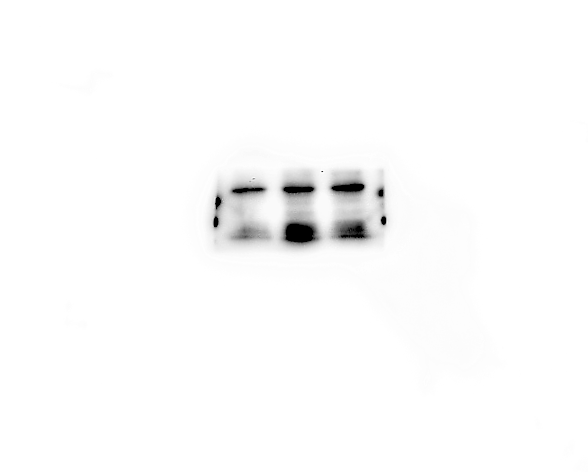

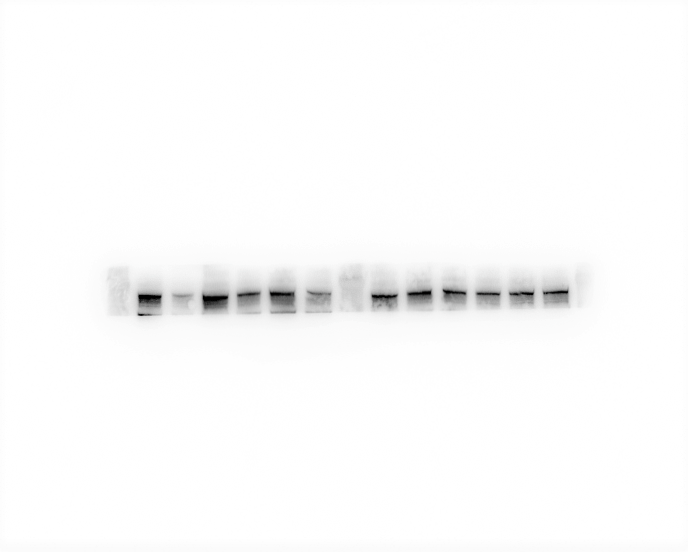


-20kda

-20kda

-15kda

CDK4 (Fadu sh-FOXD1-NC, sh-FOXD1#1, sh-FOXD1#3); CDK4 (Vector, OE)


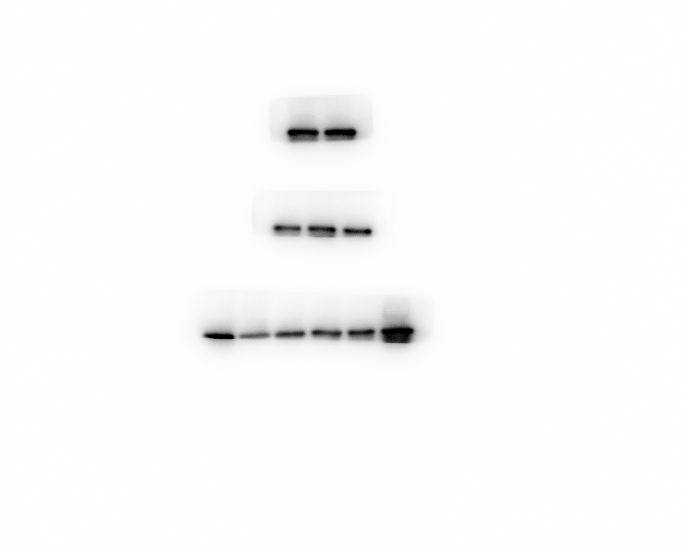

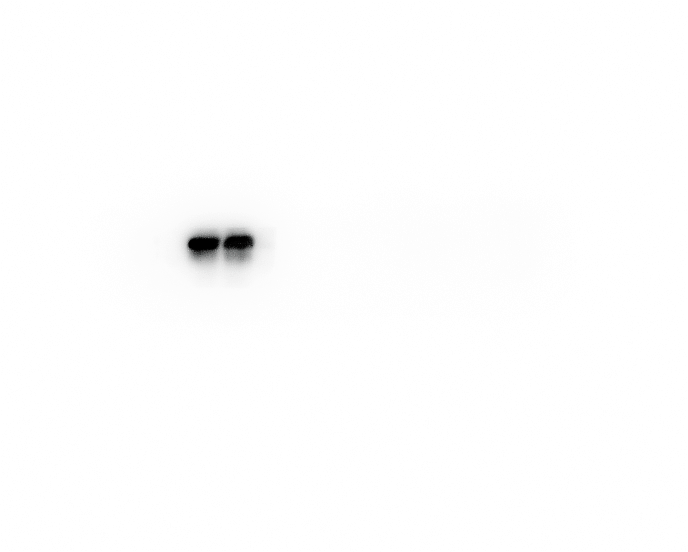


-35kda

-35kda

p-CDK4 (Fadu sh-FOXD1-NC, sh-FOXD1#1, sh-FOXD1#3); p-CDK4 (Vector, OE)


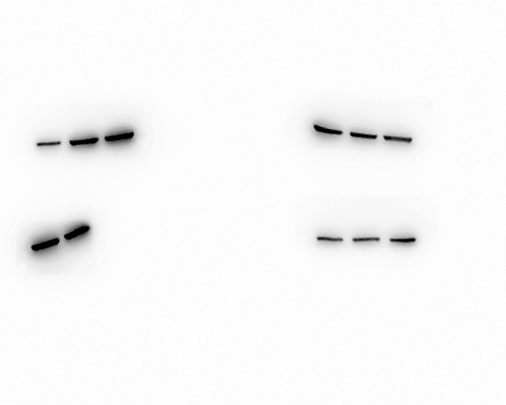

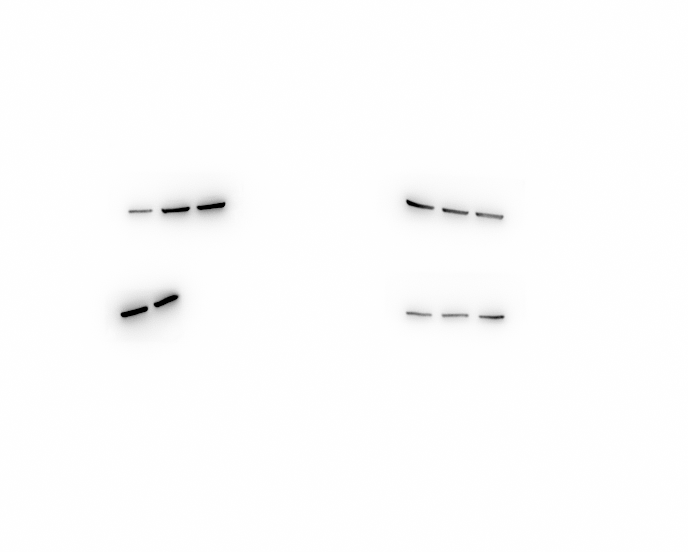


-35kda

-35kda

CDK6 (Fadu sh-FOXD1-NC, sh-FOXD1#1, sh-FOXD1#3); CDK6 (Vector, OE)


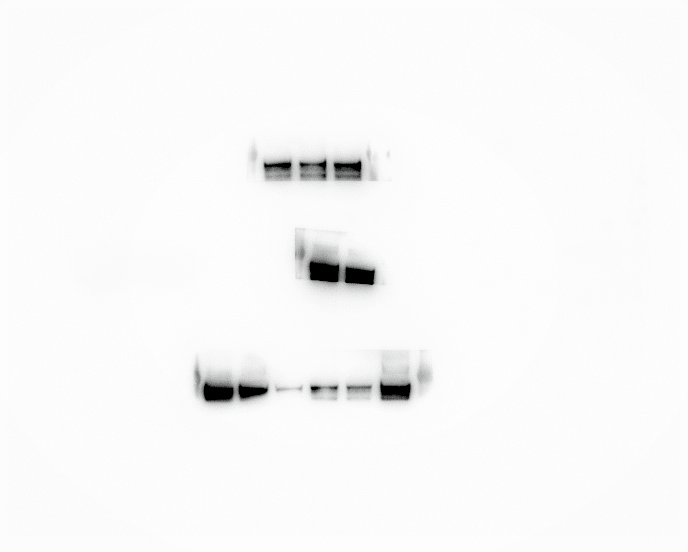

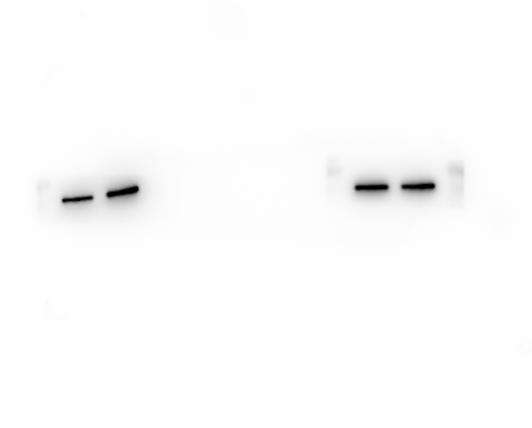


-35kda

-35kda

p-CDK6 (Fadu sh-FOXD1-NC, sh-FOXD1#1, sh-FOXD1#3); p-CDK6 (Vector, OE)


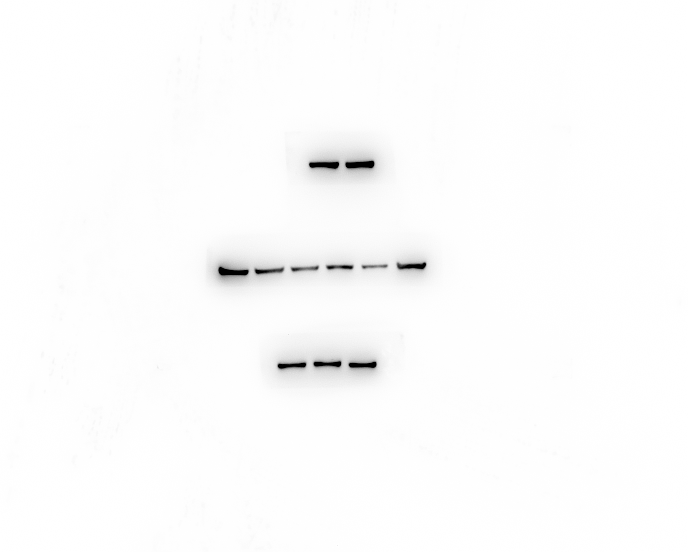

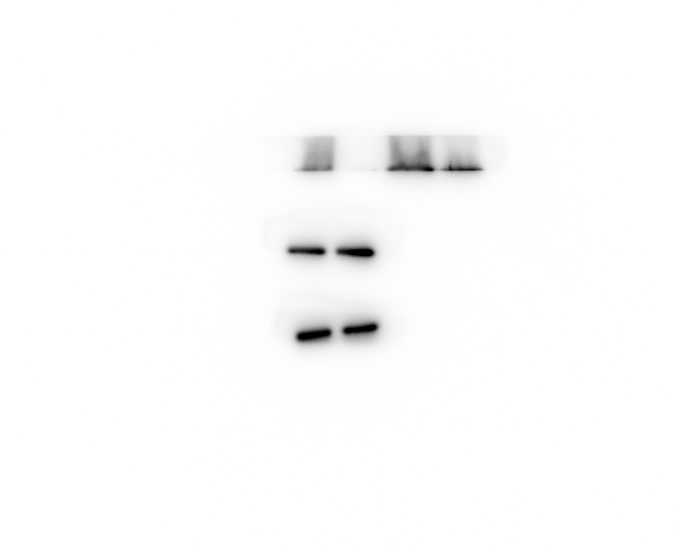


-35kda

-35kda

CDK2 (Fadu sh-FOXD1-NC, sh-FOXD1#1, sh-FOXD1#3); CDK2 (Vector, OE)


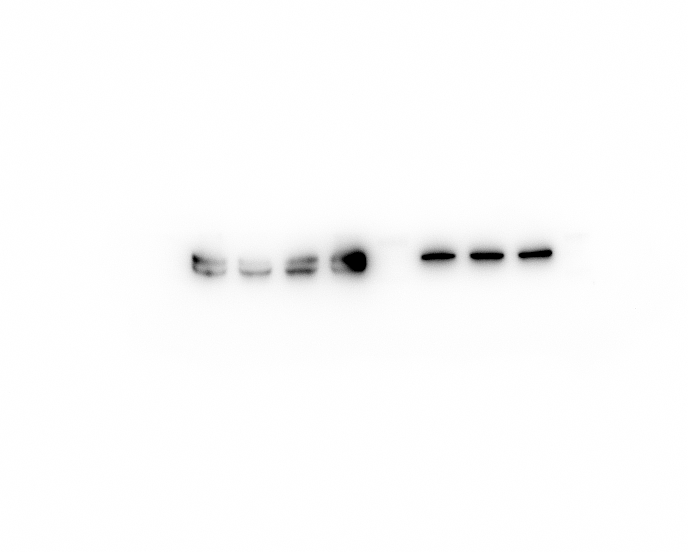

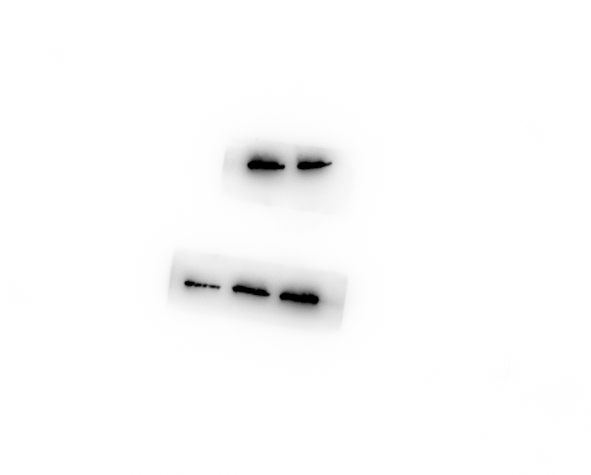


-35kda

-35kda

p-CDK2 (Fadu sh-FOXD1-NC, sh-FOXD1#1, sh-FOXD1#3); p-CDK2 (Vector, OE)


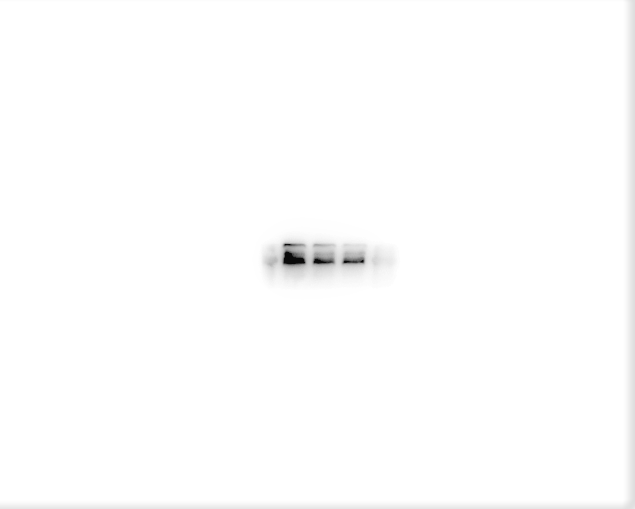

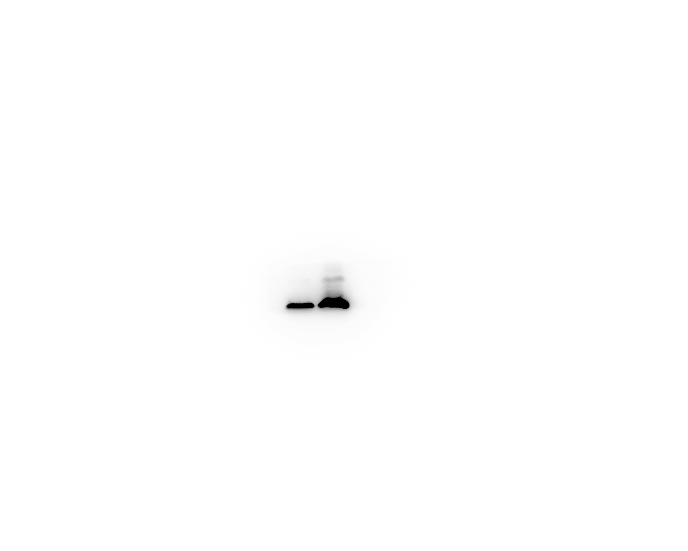


-35kda

-35kda

cyclinE1 (Fadu sh-FOXD1-NC, sh-FOXD1#1, sh-FOXD1#3); cyclinE1 (Vector, OE)


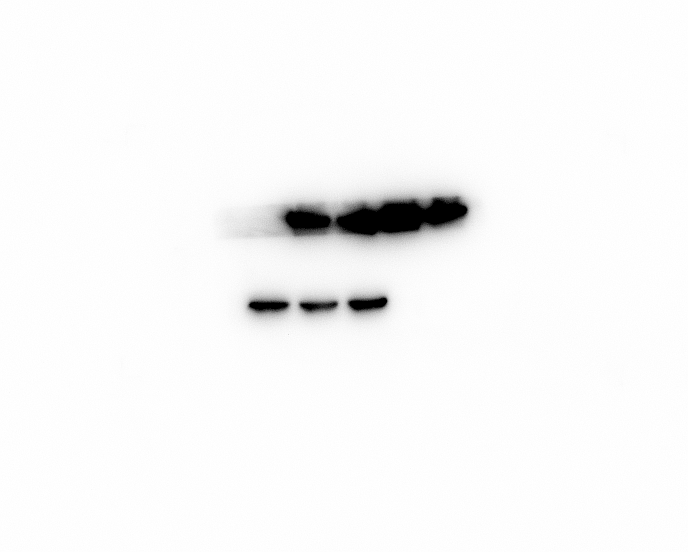

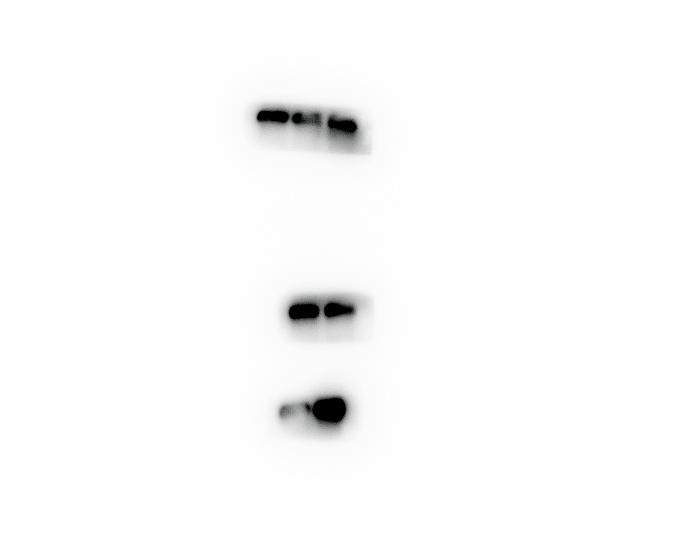


-45kda

-45kda

Rb (Fadu sh-FOXD1-NC, sh-FOXD1#1, sh-FOXD1#3); Rb (Vector, OE)


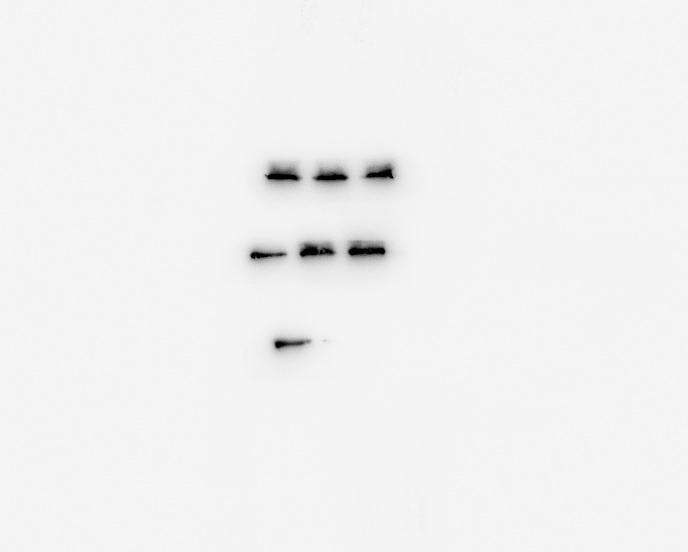

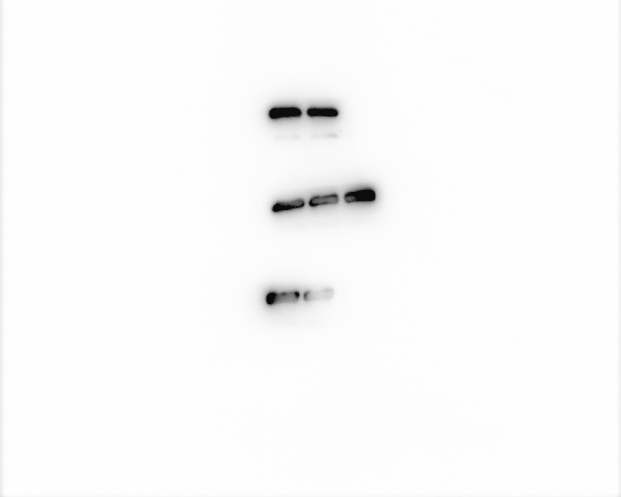


-100kda

-100kda

p-Rb (Fadu sh-FOXD1-NC, sh-FOXD1#1, sh-FOXD1#3); p-Rb (Vector, OE)


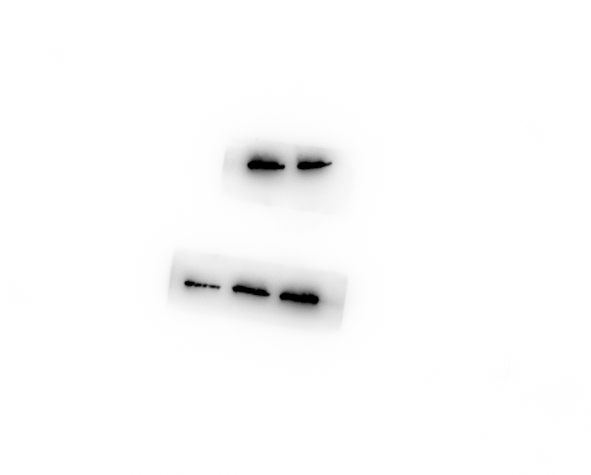

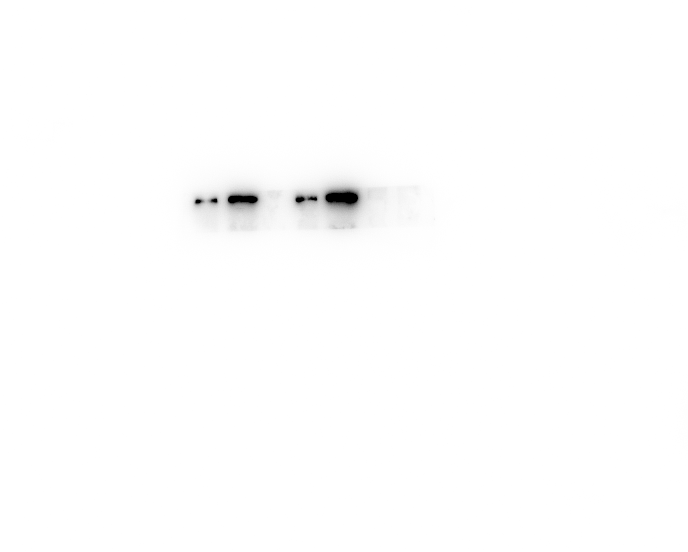


-100kda

-100kda

a-tublin (Fadu sh-FOXD1-NC, sh-FOXD1#1, sh-FOXD1#3); a-tublin (Vector, OE)


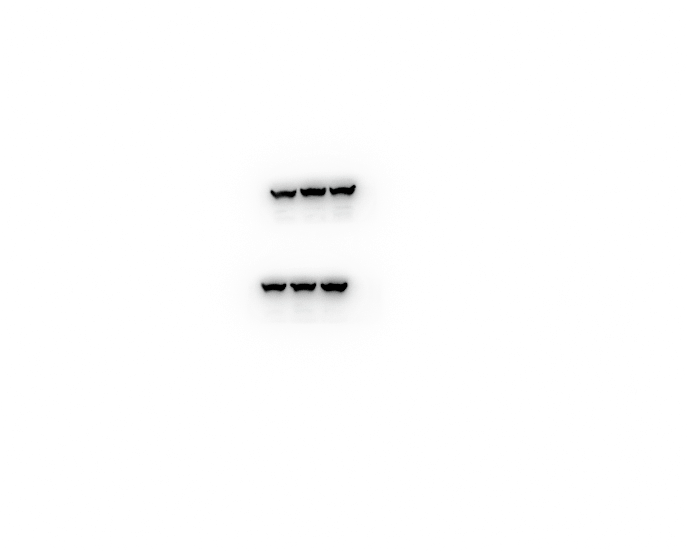

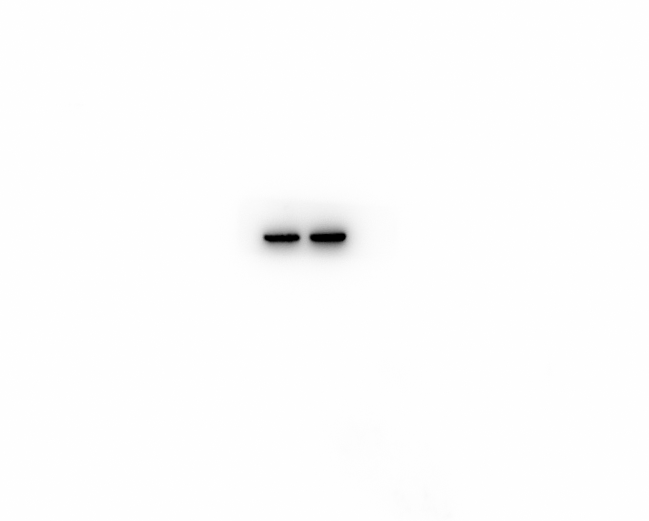


-45kda

-45kda

***Figure 7***

***Figure 7E***

FOXD1 p21


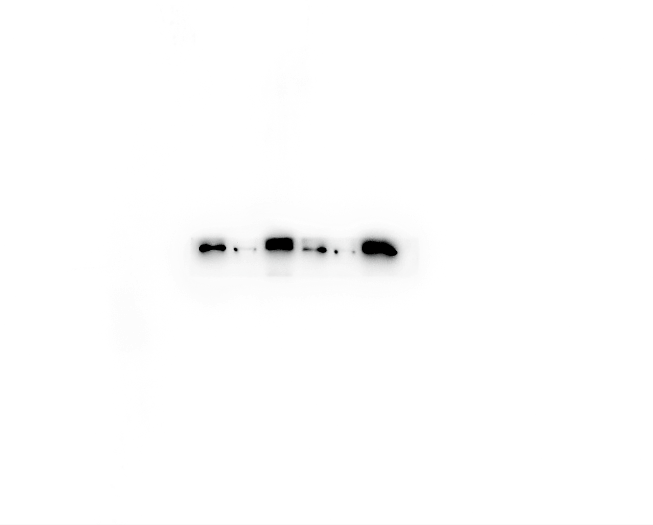

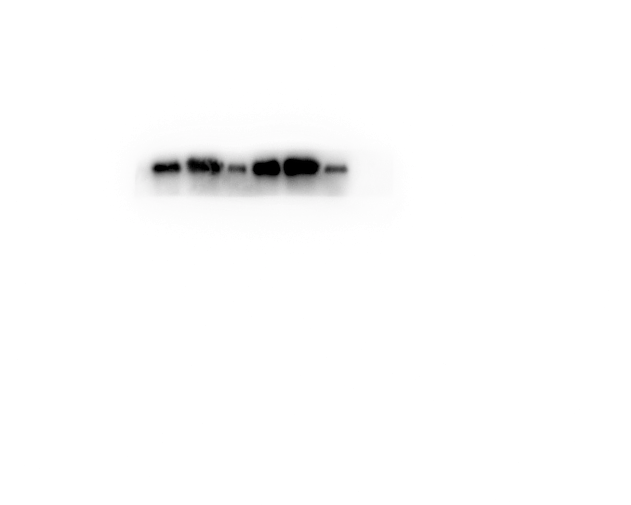


-20kda

-45kda

p-CDK2 p-Rb


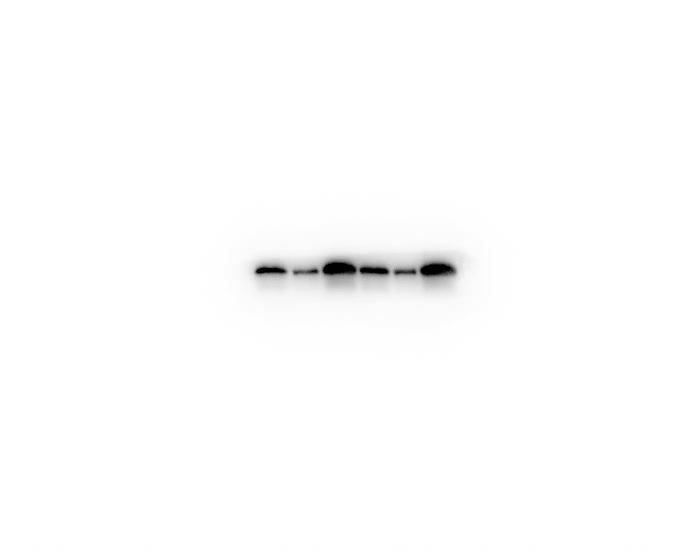

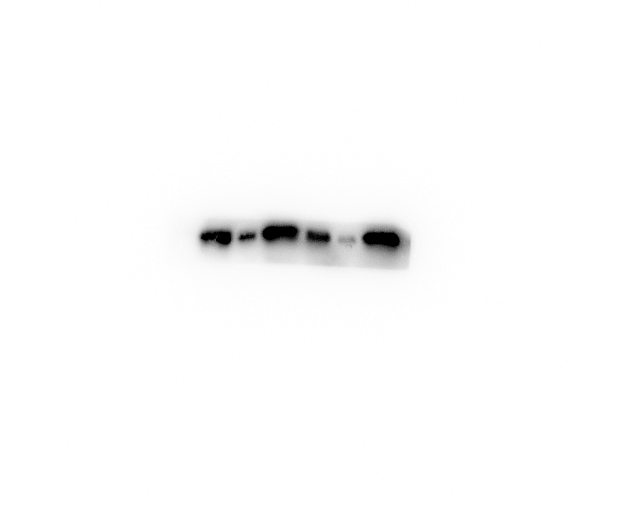


-100kda

-35kda

a-tublin


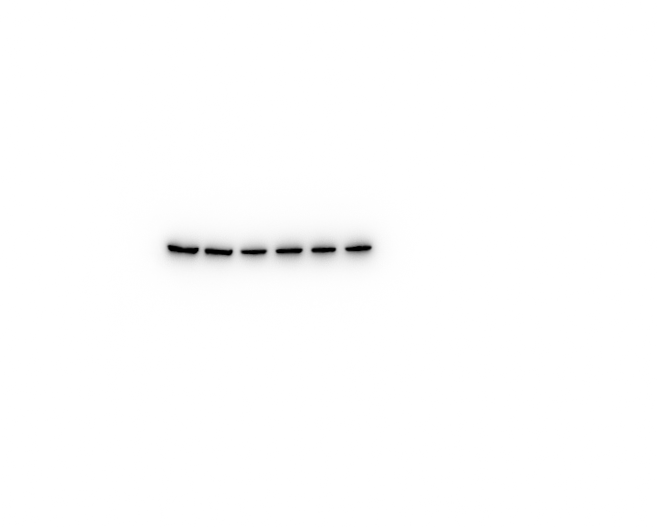


-45kda
